# Supplementary figures and images for: Global implementation survey of Integrated Management of Childhood Illness (IMCI): 20 years on
Source: BMJ Open. 2018 Jul 30;8(7):e019079. doi: 10.1136/bmjopen-2017-019079 (PMC6067364; doi:10.1136/bmjopen-2017-019079)

*Flow diagram*

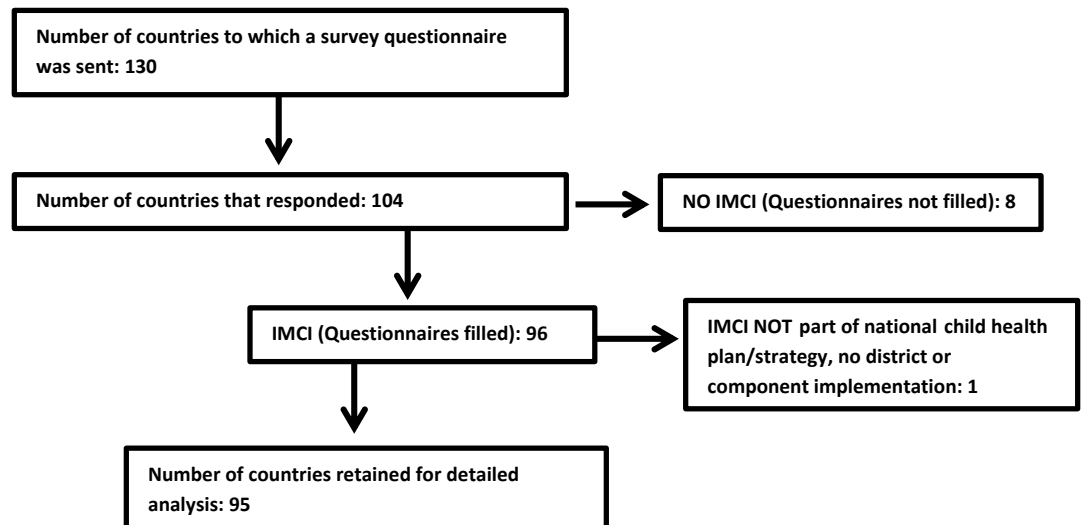

Supplement: Supplementary file 1 [file bmjopen-2017-019079supp001.pdf]
