# Supplementary material for: Global implementation survey of Integrated Management of Childhood Illness (IMCI): 20 years on
Source: BMJ Open. 2018 Jul 30;8(7):e019079. doi: 10.1136/bmjopen-2017-019079 (PMC6067364; doi:10.1136/bmjopen-2017-019079)
Supplement: Supplementary file 2 [file bmjopen-2017-019079supp002.pdf]

Web Appendix

Table 1. Response Rate to IMCI Global Implementation Survey

WHO Region	Total number of WHO MS	Member States (N) to which a survey questionnaire was sent	WHO MS that responded*	WHO MS that reported no IMCI (did not participate in the survey)	WHO MS that responded IMCI not in national plan/no district implementing nor component implemented	Response rate
African	47	47	45	4		95.7
Americas	35	33	19	4		57.6
Eastern Mediterranean	21	16	14			87.5
Europe	53	7	6			85.7
South-east Asia	11	11	9			81.8
Western Pacific	27	16	11		1	68.8
**Total**	**194**	**130**	**104**	**8**	**1**	80.0

MS = Member State; * Includes those MSs that responded not having adopted IMCI and therefore not answering the questions of the survey

Table 2. Respondents to IMCI Global Implementation Survey by WHO Region

WHO Region	Questionnaires included in final analysis	Three-team response (MoH/ WHO/ UNICEF)	Response by MoH/ WHO team	Response by MoH alone	Response by WHO alone	Response by WHO/ UNICEF team	Unknown
African	41	1	1	22	14	-	3
Americas	15	-	5	9	1	-	-
Eastern Mediterranean	14	3	7	4	-	-	-
Europe	6	1	-	3	1	1	-
South-east Asia	9	-	-	6	3	-	-
Western Pacific	10	-	1	9	-	-	-
**Total**	**95**	**5**	**15**	**52**	**19**	**1**	**3**
