# Supplementary material for: Global implementation survey of Integrated Management of Childhood Illness (IMCI): 20 years on
Source: BMJ Open. 2018 Jul 30;8(7):e019079. doi: 10.1136/bmjopen-2017-019079 (PMC6067364; doi:10.1136/bmjopen-2017-019079)
Supplement: Author's manuscript [file bmjopen-2017-019079.draft_revisions.pdf]

BMJ Open

BMJ Open is committed to open peer review. As part of this commitment we make the peer review history of every article we publish publicly available.

When an article is published we post the peer reviewers' comments and the authors' responses online. We also post the versions of the paper that were used during peer review. These are the versions that the peer review comments apply to.

The versions of the paper that follow are the versions that were submitted during the peer review process. They are not the versions of record or the final published versions. They should not be cited or distributed as the published version of this manuscript.

BMJ Open is an open access journal and the full, final, typeset and author-corrected version of record of the manuscript is available on our site with no access controls, subscription charges or pay-per-view fees (http://bmjopen.bmj.com).

If you have any questions on BMJ Open's open peer review process please email info.bmjopen@bmj.com

BMJ Open

Global implementation survey of the Integrated Management of Childhood Illness (IMCI) – twenty years on

Journal:	*BMJ Open*
Manuscript ID	bmjopen-2017-019079
Article Type:	Research
Date Submitted by the Author:	09-Aug-2017
Complete List of Authors:	Boschi-Pinto, Cynthia; WHO, Maternal, Newborn, Child and Adolescent Health; Universidade Federal Fluminense, Instituto de Saúde Coletiva<br>Labadie, Guilhem; WHO, Maternal, Newborn, Child and Adolescent Health<br>Dilip, Thandassery; World Health Organization, Maternal, Newborn, Child and Adolescent Health;<br>Oliphant, Nicholas; The Global Fund to Fight AIDS, Tuberculosis, and Malaria (Geneva, Switzerland), Public Health Monitoring and Evaluation ; UNICEF, NY<br>Dalglish, Sarah; World Health Organization, Maternal, Newborn, Child and Adolescent Health<br>Aboubaker, Samira; World Health Organization, Maternal, Newborn, Child and Adolescent Health<br>AGBODJAN-PRINCE, Olga; World Health Organization, Regional Office for Africa<br>Desta, Teshome; World Health Organization, Inter-country Support Team for East and Southern Africa<br>Habimana, Phanuel; World Health Organization/Regional Office for Africa,<br>Butrón-Riveros, Betzabé ; World Health Organization, Regional Office for the Americas<br>Al-Raiby, Jamela; World Health Organization, Regional Office for Eastern Mediterranean<br>Siddeeg, Khalid; World Health Organization, Regional Office for the Eastern Mediterranean<br>Kuttumuratova, Aigul ; World Health Organization, Regional Office for Europe<br>Weber, Martin; World Health Organization, Regional Office for Europe<br>Mehta, Rajesh; WHO Regional Office for South-east Asia<br>Neena, Raina; WHO Regional Office for South-east Asia<br>DAELMANS, Bernadette; World Health Organization, Chils and Adolescent Health and Development<br>Diaz, Theresa; World Health Organization, Maternal, Newborn, Child and Adolescent Health; UNICEF, NY
**Primary Subject Heading**:	Global health
Secondary Subject Heading:	Health policy, Paediatrics, Public health
Keywords:	Child Health, IMCI, Strategic Review, IMCI Global Survey

For peer review only

Global implementation survey of the Integrated Management of Childhood Illness (IMCI) – twenty years on

Authors

Cynthia Boschi-Pinto[a,b], Guilhem Labadie[a], Thandassery Ramachandran Dilip[a], Nicholas Oliphant[c,d], Sarah Dalglish[a], Samira Aboubaker[a], Adjoa Agbodjan-Prince[e], Teshome Desta[f], Phanuel Habimana[g], Betzabe Butron-Riveros[h], Jamela Al-Raiby[i], Khalid Siddeeg[i], Aigul Kuttumuratova[j], Martin Weber[j], Rajesh Mehta[k], Neena Raina[k], Bernadette Daelmans[a], Theresa Diaz[a]

[a] Department of Maternal Newborn Child and Adolescet Heath, World Health Organization
[b] Instituto de Saúde Coletiva, Universidade Federal Fluminense
[c] The Global Fund to Fight AIDS, Tuberculosis, and Malaria (current)
[d] UNICEF during the development of the survey (previous)
[e] World Health Organization, Regional Office for Africa, Ouagadougou, Burkina Faso
[f] World Health Organization, Regional Office for East and Southern Africa, Harare, Zimbabwe
[g] World Health Organization, Regional Office for Africa, Brazzaville, Congo
[h] World Health Organization, Regional Office for the Americas
[i] World Health Organization, Regional Office for Eastern Mediterranean
[j] World Health Organization, Regional Office for Europe
[k] World Health Organization, Regional Office for South-east Asia

Corresponding author: Cynthia Boschi-Pinto
World Health Organization, department of Maternal Newborn Child and Adolescent Health
Avenue Appia 20, 1211 Geneva, Switzerland
Email address: pintoc@who.int
Phone number: +41227913453
Fax number: +41227914853

For peer review only

Abstract

Objective: To present the main results of the IMCI Global Survey, carried out in April-June 2016 as part of the IMCI Strategic Review.

Setting: The 95 countries that participated in the survey are home to 82% of the global under-five population and account for 95% of the 5.9 million deaths that occurred among children less than five years of age in 2015; 93 of them are LMICs.

For peer review only - http://bmjopen.bmj.com/site/about/guidelines.xhtml

Methods: Questionnaires and data analysis focused on providing: 1) a general overview of the current level of organization and financing of IMCI at country level; 2) a description of implementation of IMCI's three original components; and 3) reported innovations, barriers and opportunities in expanding access to care for the sick child. A single data file was created using all information collected. Analysis was performed using STATA version 11.

Participants: No patient or population interviewed/examined for this analysis. Countries were the unit of analysis.

Interventions and Outcome measures: This was a survey to measure the status of IMCI implementation. No intervention or outcome measures included in this study.

Results: Eighty-one percent of countries reported that IMCI implementation encompassed all three components. Almost half (46%) reported implementation in 90% or more districts as well as all three components in place. Despite these high reported implementation rates, the strategy is not reaching the children who need it most as implementation is lowest in high mortality countries (39%; 7/18), where it is probably most needed.

Discussion: This survey is a unique opportunity to better understand how and in which direction the implementation of IMCI has evolved over 20 years since its inception. Results obtained are extremely informative and should be used to assist in formulating strategies, policies and activities to support improvements in health and survival of children and to help achieving the UN health-related, post-2015 SDGs.

Article Summary

Strengths and limitations of this study

This article summarizes the information obtained from the *first survey assessment of the status of IMCI implementation in 95 responding countries worldwide*, twenty years after its launching in the mid-1990s

This survey is a unique opportunity to better understand how and in which direction the implementation of IMCI has evolved over 20 years since its inception and to help steer future child health directions

Limitations are mostly due to the study design: respondents may not feel comfortable providing answers that present the country in an unfavourable manner and therefore may not provide accurate answers.

The WHO African region is likely to be overrepresented in the survey responses, but disaggregation by mortality level and income groups reduces the effect of this overrepresentation on the interpretation of results.

For peer review only - http://bmjopen.bmj.com/site/about/guidelines.xhtml

Introduction

As part of the Strategic Review of the Integrated Management of Childhood Illnesses (IMCI) (1,2), a global survey was carried out in April – June 2016 to assess the extent to which this strategy has been adopted and scaled up in countries over the past two decades. Much work has been done on different aspects of this strategy's implementation and effectiveness over the past 20 years, but to date there has been no holistic description of IMCI implementation worldwide. The referred survey attempts to fill that gap. This article reports on its main results and provides an overview of the current status of global implementation of IMCI as informed by countries. The article also presents the main strengths and barriers in IMCI implementation, as stated by respondents, as well as suggested ways forward.

Methods

The survey instrument was developed by WHO with inputs from UNICEF and was tested in three different WHO regions to ensure questions were clear and appropriate and to estimate the time needed to complete the questionnaire. The final version was made available in all six UN languages (Arabic, Chinese, English, French, Spanish and Russian). Questions and data analysis focused on providing: 1) a general overview of the current level of organization and financing of IMCI at country level; 2) a description of implementation of IMCI's three original components and of some additional components, such as iCCM; and 3) the main innovations, barriers and opportunities used in expanding access to care for the sick child. Questionnaire instructions asked that responses be provided by in-country teams consisting of representatives of the ministry of health (MoH) and country offices of WHO and UNICEF. Questionnaires included mostly closed-ended questions. Countries identified the principal strengths of IMCI and barriers to its implementation by agreeing or disagreeing with a suggested list, and answered open-ended questions about future actions to sustain and improve implementation. Data analysis was disaggregated by levels of under-five mortality rates (U5MRs), gross national income (GNI) per capita, and extent of IMCI implementation. Information on under-five mortality (numbers and rates) for 2015 was obtained from the United Nations Inter-agency Group for Child Mortality Estimation (UN-IGME) report on levels and trends in child mortality (3). Countries were categorized into low (<40 deaths per 1000 live births), middle (40-79 deaths per 1000 live births) and high mortality (80+ deaths per 1000 live births). Countries were also classified by income level (low, middle, and high) according to current GNI per capita (2016) using World Bank data (4), and as high (>90% of districts reported to have implemented IMCI), middle (50-90% of districts reported to have implemented IMCI) and low implementers (<50% of districts reported to have implemented IMCI), according to the proportion of districts reported to have implemented the strategy. Information on under-five population for the year 2015 was obtained from the UN Population Division (5). A single data file was created using all information collected. Analysis was performed using STATA version 11. More detailed methods are described elsewhere (6).

Patient Involvement

This is a key-informant survey carried out at national level and no individual patient data was included.

For peer review only - http://bmjopen.bmj.com/site/about/guidelines.xhtml

Results

General

Questionnaires were sent to a total of 130 WHO Member States with a response rate of 80% [Web Table 1]. Of the 104 countries that responded to the survey, eight reported no IMCI in the country, leaving 96 countries for the general analysis. One country was dropped from the analysis because IMCI was not reported to be part of their national child health plan or strategy, no district was reported to be implementing IMCI and none of the three components was reported to be in place (Figure 1).

Of the 95 countries retained for final analysis (Figure 2), only two (Oman and Uruguay) are classified as high-income countries according to the World Bank; 64 are classified as middle-income and 29 as low-income. The instruction for questionnaires to be filled in by a three in-country team was followed in only five countries. In the majority of countries only the MoH provided answers [Web Table 2]. For three countries, the information on respondents is not available.

Eighty-nine per cent (82/94) of countries had an IMCI focal point at national level, whereas only 68% (62/91) had one at the sub-national (regional or district) level. Among high mortality countries, 88% (17/20) had a national focal point, but less than half this proportion (42%; 8/19) had subnational focal points. The presence of regional/district focal point was much higher in countries with U5MRs below 80 per 1000 live births (75%; 54/72) than in countries with higher rates. The government is generally reported to be the principal funding source for IMCI expenditures at first level health facilities and in the community, except for the training courses and per diem. For the latter, donors constituted the main sources of funding. In Figure 3 we show the proportion of countries reporting government as primary funder of different elements of IMCI in first level health facility by under-five mortality level.

Implementation of IMCI and its three components

For the purpose of the survey, IMCI is said to be implemented if it is included in national and district work plans and budgets, if district health facilities use the strategy to provide care for the sick child (with or without community-based IMCI), and if providers are trained in IMCI to care for the sick child at national and district levels. Of the 95 countries included in the full analysis, 81 responded to the question on the proportion of districts implementing IMCI: 51 (63%) countries reported implementing IMCI in all districts; 54 (67%) in 90% or more districts and 62 (77%) countries reported implementing it in at least three quarters of their districts. Implementation in more than 90% of districts is lowest in high mortality countries (39%; 7/18). Only seven of the 54 high implementer countries (13%) were high mortality countries. One of the key indicators proposed to measure the programmatic target for coverage and performance of the strategy is the proportion of health facilities in which 60% or more of health workers involved in child care have been trained in IMCI. In most countries (58%; 43/74), half or more of the first level health facilities had at least 60% of health workers involved in child care trained in IMCI.

Figure 4 shows the proportion of countries reporting the components that had been encompassed during IMCI implementation. While 92 of the 94 responding countries reported having implemented the

For peer review only - http://bmjopen.bmj.com/site/about/guidelines.xhtml

first component of IMCI (improving health worker skills), 89 reported implementing the second (strengthening health systems), and 78 responded having implemented the third (improving family and community practices).

First component – improving health worker skills

The original training course for health workers lasted 11 days, with 30% of time spent in clinical sessions (7). Most responding countries (42%; 39/92) used shortened or abridged versions of the original course. Clinical practice was at least 25% of the training time in 79% of the countries (72/91). Nearly three quarters of countries (72%; 67/93) reported implementing some sort of IMCI pre-service training. A majority of middle and high implementer countries (81%; 54/67) reported conducting pre-service training, whereas 46% (6/13) of the countries with fewer than 50% of districts implementing IMCI did so. The proportion of countries that introduced pre-service education in at least 75% of any training institutions was lowest (29%; 4/14) in high mortality countries.

Generic case management guidelines and related training materials were targeted to be appropriate in the majority of developing countries where U5MR is higher than 40 per 1000 live births, and where there is transmission of *P. falciparum* malaria (8). Over the years, IMCI chart booklets have been continuously adapted to the context and changing scenarios of each country. Since the last global update in 2014, 81% (69/85) of countries reported having updated their national chart booklets. Recognizing the increased importance of the burden of newborn mortality, nearly all countries (95%; 89/94) have adapted IMCI guidelines to include care of the sick newborn in the first week of life. Development or revision of national treatment guidelines based on the 2013 revision of the WHO Pocket Book of Hospital Care for Children and its adaptation in referral facilities were reported by 55% (48/87) and 44% (39/88) of responding countries, respectively. Considering Emergency Triage Assessment and Treatment (ETAT), 23% of the 65 responding countries reported introducing them in at least 75% of hospitals. This proportion is remarkably lower (17%) in the 41 middle and high mortality countries than in the 24 low mortality countries (38%).

The most common conditions added to the guidelines in national adaptations were jaundice in newborn babies and young infants (77%; 72/93), HIV (63%; 59/93), sore throat (59%; 55/93), and skin conditions (44%; 41/93). Other illnesses frequently included are tuberculosis and dengue haemorrhagic fever. Adaptations varied according to regional or national epidemiological profile.

Second component – strengthening health systems

Improving and sustaining the quality of paediatric care is an integral part of strengthening health systems. An assessment of quality of paediatric hospital care was conducted in 42% (39/92) of responding countries. Fifty-two per cent (34/65) of middle and high implementer countries reported having such assessment compared to only 8% (1/13) of low implementer countries. There were no important differences in assessments of quality of care (QoC) between countries with high or low mortality rates. Fifty eight per cent of the 92 responding countries reported having a paediatric QoC improvement programme for health facilities in their MoH. Countries that report improvement programmes account together for nearly 3 million (50%) of all 5.9 million under-five deaths that occurred worldwide in 2015.

For peer review only - http://bmjopen.bmj.com/site/about/guidelines.xhtml

Supervision and monitoring are key factors for sustainable QoC and for strengthening the health system. Few countries (15%; 10/66) reported more than 75% of first level facilities with at least one supervisory visit in the last six months; half (33/66) had less than one quarter of these facilities receiving such visits. In general, monitoring of IMCI implementation has been carried out infrequently. Only 33% of countries (30/91) reported having a comprehensive IMCI monitoring and evaluation plan. Fifteen per cent (3/20) of high mortality countries reported having such a plan, while this proportion was 38% (27/71) in middle and low mortality countries. Still, more than two thirds of countries (70%; 66/94) reported that their Health Management Information System (HMIS) includes monitoring indicators for IMCI. Most of these indicators are collected at the primary health care level (83%; 54/65); only four countries reported having monitoring indicators for IMCI collected at community level.

In 2009, WHO launched the "Managing Programmes to Improve Child Health" course, which has been introduced in almost half (48%; 42/88) of responding countries. The number of such trained managers per under-five population varies widely, ranging from 1 manager per 840 thousand under-five children in Lao PDR to 1 manager for 1500 children in Kiribati – a more than 500-fold difference. Tools for bottleneck analysis and strategic planning have been introduced in 33 of the 79 responding countries (42%).

The main innovations reported by countries are related to information communication technologies (ICTs), which include eHealth, mHealth and Rapid SMS. Use of such innovations in the implementation of IMCI was reported over six categories: data collection and reporting (70%; 60/86); supply chain management (58%; 51/88); behaviour change communication (54%; 48/89); health provider training and education (54%; 46/85); electronic decision support (49%; 42/85); and financial transactions and incentives (41%; 35/86).

Third component - Improving family and community practices

Improving family and community practices was reported as part of IMCI implementation by 83% (78/94) of responding countries (Figure 4). Eighty-nine per cent (48/54) of medium and high mortality countries report implementation of this component, while 75% (30/40) of low mortality countries reported implementing it. In general, activities to *promote key family practices for child health* have been implemented through home visits, and through social mobilization or community groups. The main delivery mechanisms used are illustrated in Figure 5. The majority of responding countries (71%; 45/63) reported that one community health worker (CHW) covered up to 1000 under-five population. Supervision for CHWs was mainly provided by IMCI trained supervisor (63%; 58/92) and through mentorship in health facilities (45%; 41/92), followed by non-IMCI trained supervisor (27%; 25/92), and peer-support (15%; 14/92).

Care for children under five years of age was reported to be provided by more than three quarters (78%, 72/92) of responding countries. The implementation of community case management (CCM), in general, was higher in high (85, 17/20) and medium (94%, 31/33) mortality countries as compared to low mortality countries (62%, 24/39). These variations are similar to those observed for the presence of CCM policy in countries. Among the 72 countries that reported that CHWs provide care for children, 93%

For peer review only - http://bmjopen.bmj.com/site/about/guidelines.xhtml

(67 countries) reported having CCM for diarrhoea. Amid these 67 countries, all but one responded to the corresponding question on policy and 80% (53/66) reported having the relevant written policy in place. Conversely, four countries reported having the policy in place but not implementing it. In contrast to the 93% of countries that reported providing CCM for diarrhoea, only 74% (53/72) reported that CHWs provide CCM for pneumonia. From these 53 countries, 43 reported having the relevant written policy in place. Of the 66 malaria-endemic countries that responded to the question, 89% (59/66) reported CCM for malaria by CHWs; all 27 low income countries implemented it. Other aspects of community care, also supported by WHO and UNICEF, include the management of severe acute malnutrition (SAM) (9) and home visits (10). CCM for SAM was mentioned by 85% (61/72) of countries. More countries reported providing home visits to newborn babies (91%, 59/65) than for children beyond the newborn period (76%; 55/72).

The provision of timely and effective *integrated treatment* of diarrhoea, pneumonia and malaria – integrated community case management (iCCM) (11) – by CHW to populations with limited access to facility-based health care providers was reported by 64% (46/72) of responding countries. Among the 66 malaria-endemic countries that responded to this question, the same proportion (64%; 42/66) reported existence of iCCM. Policies were present in 34 of these 42 countries (81%).

Strengths, Barriers, and Ways forward

The major strengths of IMCI most frequently identified by 93 responding countries were: the holistic approach to the child (90%; 84 countries), the rational use of medicine (89%; 83 countries), the quality of health services (87%; 81 countries), and the efficiency of service provision (80%; 74 countries).

At national level, the reported major barriers to implementing IMCI were budget for training (85%; 78/92), mentorship and supervision (74%; 68/92), cost or sustainability of activities (63%; 58/92), and availability of dedicated budget line (60%; 55/92). Major barriers reported at regional/district level were staff turnover (84%; 76/91), budget for training (82%; 75/91), and mentorship and supervision (74%; 67/91). Barriers reported at facility level were staff retention (80%; 73/91), mentorship and supervision (79%; 72/91), and staff motivation (74%; 67/91). High mortality and/or low income countries were more likely to report barriers related to budget for training, and medicine procurement and supply chain at national level. High implementer countries were more likely to report issues around mentorship and supervision, and less likely to report barriers on programme management and on political support and ownership.

When asked to suggest ways to strengthen child health services and make IMCI more relevant to today's needs, countries commonly advocated for better integration of current strategies into general health services, with a costed work plan and committed budget, as well as improved training at all levels, including pre-service training, and supportive supervision and mentoring. Health systems issues were said to limit implementation, with a strong need for a functioning supply chain, HMIS and monitoring and evaluation system. Better use of communications technologies was also suggested. Furthermore, compliance with national guidelines and good implementation practices should be recognized and rewarded, respondents said. Community-level activities including outreach and home visits need more

For peer review only - http://bmjopen.bmj.com/site/about/guidelines.xhtml

emphasis, as do the management of sick newborns at all levels. At policy level, respondents said strategic planning, improved internal coordination (via a dedicated focal person for child health, regular review meetings, national steering committee) and effective coordination with partners to overcome fragmentation would be essential. To achieve these goals, high-level advocacy and political support is said to be required, ensuring government leadership and ownership.

Discussion

This article summarizes the information obtained from the first survey assessment of the status of IMCI implementation in 95 responding countries worldwide, twenty years after its launching in the mid-1990s (6). In 1998, 12 countries had already moved into a phase of expansion of IMCI's first component and had begun introducing the strategy's other two components (12). Twenty years later, coverage of IMCI is reported to be comprehensive in many target countries: at least two-thirds of the 95 countries that responded to the survey questionnaire reported implementation in 90% or more districts in 2016. However, although many countries have expanded aspects of implementation to a large proportion of their districts, few have achieved full scale up and, in many aspects, implementation remains incomplete. Moreover, despite the high reported implementation rates, the strategy is still not reaching the strategy is not yet reaching the children who need it most - coverage of IMCI is lowest in high mortality countries.

One of the factors originally identified by early reviewers of IMCI as influencing the general success of IMCI was the establishment of a management structure, such as a working group or task force. Early experience also suggests that countries needed to identify a coordinator for the management structure (13). At district level, a focal person for IMCI would coordinate with the central level, including the IMCI management structure and its subgroups (7). As countries move towards decentralization, the presence of sub-national focal points increases in importance. Our results show a higher proportion of countries with an IMCI focal point at national level than at the sub-national (regional or district) level.

There is widespread recognition that IMCI will only result in improvements in child health and survival if training activities are accompanied by effective efforts to strengthen health systems and reach children and mothers in the community. Our findings of very high general implementation rates of the first and second components, in detriment of the third component as well as the low coverage of specific aspects of the second component demonstrate that this full implementation scenario is yet to be achieved. Nevertheless, it is worth noting that high implementer countries (≥90% of districts) with all three IMCI components in place are home for 160 million of the global under-five children. Also of note is the fact that among the 24 low and lower-middle income countries that have achieved MDG's 4 target, 18 (75%) are part of these high implementer countries with all three components in place.

The training course for health workers developed by WHO and UNICEF is one of the key elements of IMCI strategy. Some limited evidence suggest the original 11-day training course to be more effective than shortened training (14); however, concerns regarding the costs and the absence of health workers from the services have been raised (15). Most countries reported using shortened or abridged versions of the original course. Flexibility to add conditions according to regional and national epidemiologic

For peer review only - http://bmjopen.bmj.com/site/about/guidelines.xhtml

profile has facilitated adaptation of IMCI guidelines to a changing epidemiological profile. Supervision is the weakest area under the second IMCI component, with only 15% of countries reporting more than three-quarters of first level facilities with at least one supervisory visit in the 6 months before the survey. In general, activities to *promote key family practices for child health* have been implemented through home visits, and through social mobilization or community groups.

In 2012, the key elements of CCM were brought together in a package - iCCM (11), typically delivered by CHWs at the community level. It encompasses treatment for (i) childhood pneumonia with antibiotics, (ii) diarrhoea with zinc and oral rehydration salts and (iii) malaria with artemisinin combination therapy. iCCM was reported to be implemented in two-thirds of responding countries, although policy was present in 78% of them. The limiting factor was CCM for pneumonia.

The most commonly mentioned barriers have their roots in insufficient funding and weak health systems. Nevertheless, the major strengths identified - the holistic approach to the child, the rational use of medicine, the quality of health services, and the efficiency of service provision, and suggested ways forward offer important instruments to those thinking on and re-designing the future of child health and development.

This study has some limitations, most of which inherent to survey design. Firstly, respondents may not feel comfortable providing answers that present the country in an unfavourable manner and therefore may not provide accurate answers. Also, respondents may not always be fully aware of the situation in the country. To minimize this limitation, we have provided clear instructions for questionnaires to be responded by a three in-country team, but only five countries followed this instruction. Another 16 countries had questions answered by a two-respondent team. Bias due to non-response may also have occurred as respondents who chose to answer certain questions may differ from those who chose not to respond. Finally, the WHO African region is likely to be overrepresented in the survey responses, with 43% of the countries while the European region represents six per cent of them. Disaggregation by mortality level and income groups reduces the effect of this overrepresentation on the interpretation of results. Population wise, South-east Asian (32%), African (28%) and Western Pacific (20%) children are those mostly covered.

Despite these limitations, this survey is a unique opportunity to better understand how and in which direction the implementation of IMCI has evolved over 20 years since its inception. It is also a unique opportunity to help steer future child health directions. The 80% response rate obtained reveals the interest IMCI still elicits, especially in LMICs, and is an indicator that it should be strengthened and properly re-designed to attend the call for “Survive, Thrive, Transform” from the Global Strategy for Women’s Children’s and Adolescents’ Health 2016-2030 (16). The 95 countries that participated in this Global Survey are home to 82% of the global under-five population and account for 95% of the 5.9 million deaths that occurred among children less than five years of age in 2015. These results are therefore extremely informative and should be used to assist in formulating strategies, policies and activities to support improvements in health and survival of children and to help achieving the UN health-related, post-2015 Sustainable Development Goals (SDGs).

For peer review only - http://bmjopen.bmj.com/site/about/guidelines.xhtml

Ackowledgements

We thank our colleagues from WHO and UNICEF Country Offices and those from the Ministries of Health for their contribution on responding to the survey questionnaires and on providing follow-up of the survey.

Disclaimer

The authors* are staff members of the World Health Organization. The authors alone are responsible for the views expressed in this publication and they do not necessarily represent the decisions or policies of the World Health Organization.

Competing Interest

All authors have completed the Unified Competing Interest form (available on request from the corresponding author) and declare: no support from any organisation for the submitted work; no financial relationships with any organisations that might have an interest in the submitted work in the previous three years, no other relationships or activities that could appear to have influenced the submitted work.

Copyright

The Corresponding Author has the right to grant on behalf of all authors and does grant on behalf of all authors, an exclusive licence (or non exclusive for government employees) on a worldwide basis to the BMJ Publishing Group Ltd to permit this article to be published in BMJ editions and any other BMJPGL products and sublicences such use and exploit all subsidiary rights, as set out in our licence.

Funding

The Strategic Review of IMCI and iCCM was funded by the Bill and Melinda Gates Foundation; the authors donated their time to writing the article; the publication fees for the supplement were funded by the Health Systems Research Unit, South African Medical Research Council.

.

Contributors and sources

The authors bring a combination of expertise in epidemiology, demography, child health interventions and policies, public health, and advocacy. CBP conceptualized and wrote the first draft of the paper. GL, TRD and NO performed the analysis. GL conceptualized and performed the illustrations and revised the first draft. SD analyzed the qualitative information and wrote the last session of the paper (Strengths, barriers and ways forward). AA-P, TD, PH, BB-R, JA-R, KS, AK. MW, RM, and NR organized, tested, followed-up and reviewed the questionnaires sent to countries. All authors contributed intellectual content and critical revisions to the manuscript. All authors approved the final manuscript. CBP is guarantor.

Data Sharing

All data will be available in public domain [IMCI Global Implementation Survey Report at: http://who.int/maternal_child_adolescent/documents/imci-global-survey-report/]

For peer review only - http://bmjopen.bmj.com/site/about/guidelines.xhtml

For peer review only

For peer review only - http://bmjopen.bmj.com/site/about/guidelines.xhtml

References

(1) WHO. “Towards a Grand Convergence for child survival and health: A strategic review of options for the future building on lessons learnt from IMNCI.” Geneva: WHO, 2016. Available at: http://www.who.int/maternal_child_adolescent/documents/strategic-review-child-health-imnci/en/

(2) Dalglish et al. Methods for the Strategic Review of IMNCI and iCCM programmes BMJ 2017 (paper 2 in the supplement – TBD)

(3) WHO/ The World Bank/ United Nations Population Division. Levels & Trends in Child Mortality Report 2015. Estimates developed by the UN Inter-agency Group for Child Mortality Estimates. Available at: http://www.who.int/maternal_child_adolescent/documents/levels_trends_child_mortality_2015/en/

(4) World Bank list of economies (December 2016) https://datahelpdesk.worldbank.org/knowledgebase/articles/906519-world-bank-country-and-lending-groups. Accessed on 26.01.2017

(5) United Nations, Department of Economic and Social Affairs, Population Division (2015). World Population Prospects: The 2015 Revision, custom data acquired via website: https://esa.un.org/unpd/wpp/Download/Standard/Population/

(6) WHO. IMCI Global Implementation Survey Report. Available at: http://who.int/maternal_child_adolescent/documents/imci-global-survey-report/

(7) WHO. IMCI Planning Guide. WHO, 1999. Available at: http://apps.who.int/iris/bitstream/10665/66720/1/WHO_CHS_CAH_99.1_eng.pdf

(8) WHO/UNICEF. IMCI Information Package, 1999. Available at: http://apps.who.int/iris/bitstream/10665/65002/4/WHO_CHS_CAH_98.1D_eng.pdf

(9) WHO (2007), Community-based management of Severe Acute Malnutrition: Joint statement by WHO, WFP and UNICEF. Malnutrition: Joint statement by WHO, WFP and UNICEF. WHO, Geneva.

(10) WHO/Unicef (2009) WHO/Unicef Joint Statement on Home Visits for the Newborn Child: A strategy to Improve Survival. WHO, Geneva.

(11) UNICEF (2012) Integrated Community Case Management: WHO/UNICEF Joint Statement. UNICEF, New York.

(12) Tulloch J. Integrated approach to child health in developing countries. Lancet 1999;354 Suppl 2;SII16-20

(13) WHO/UNICEF. IMCI Information Package, 1999. Available at: http://apps.who.int/iris/bitstream/10665/65002/3/WHO_CHS_CAH_98.1C_eng.pdf

For peer review only - http://bmjopen.bmj.com/site/about/guidelines.xhtml

(14) Rowe AK, Rowe SY, Holloway KA et al. Does shortening the training on Integrated Management of Childhood Illness guidelines reduce its effectiveness? A systematic review. *Health Policy and Planning* 2012; 27:179-93.

(15) Goga AE, Muhe LM, Forsyth K, Chopra M, Aboubaker S, Martines J, Mason EM. Results of a multi-country exploratory survey of approaches and methods for IMCI case management training. *Health Res Policy Syst* 2009; 7-18.

(16) Global Strategy for Women's, Children's and Adolescents Health 2016-2030. Available at: http://www.who.int/life-course/partners/global-strategy/en/

For peer review only

List of Figures:

Figure 1: Flow diagram

Figure 2: Survey summary map

Figure 3: Proportion of countries reporting government as primary funder of various IMCI expenditures at first level health facility by under-five mortality level

Figure 4. Implementation of the three IMCI components

Figure 5. Main delivery mechanisms used for the promotion of key family practices related to child health

For peer review only - http://bmjopen.bmj.com/site/about/guidelines.xhtml

Web Appendix

Table 1. Response Rate to IMCI Global Implementation Survey

WHO Region	Total number of WHO MS	Member States (N) to which a survey questionnaire was sent	WHO MS that responded*	WHO MS that reported no IMCI (did not participate in the survey)	WHO MS that responded IMCI not in national plan/no district implementing nor component implemented	Response rate
African	47	47	45	4		95.7
Americas	35	33	19	4		57.6
Eastern Mediterranean	21	16	14			87.5
Europe	53	7	6			85.7
South-east Asia	11	11	9			81.8
Western Pacific	27	16	11		1	68.8
**Total**	**194**	**130**	**104**	**8**	**1**	80.0

MS = Member State; ***** Includes those MSs that responded not having adopted IMCI and therefore not answering the questions of the survey

Table 2. Respondents to IMCI Global Implementation Survey by WHO Region

WHO Region	Questionnaires included in final analysis	Three-team response (MoH/ WHO/ UNICEF)	Response by MoH/ WHO team	Response by MoH alone	Response by WHO alone	Response by WHO/ UNICEF team	Unknown
African	41	1	1	22	14	-	3
Americas	15	-	5	9	1	-	-
Eastern Mediterranean	14	3	7	4	-	-	-
Europe	6	1	-	3	1	1	-
South-east Asia	9	-	-	6	3	-	-
Western Pacific	10	-	1	9	-	-	-
**Total**	**95**	**5**	**15**	**52**	**19**	**1**	**3**

For peer review only

Figure 1: Flow diagram

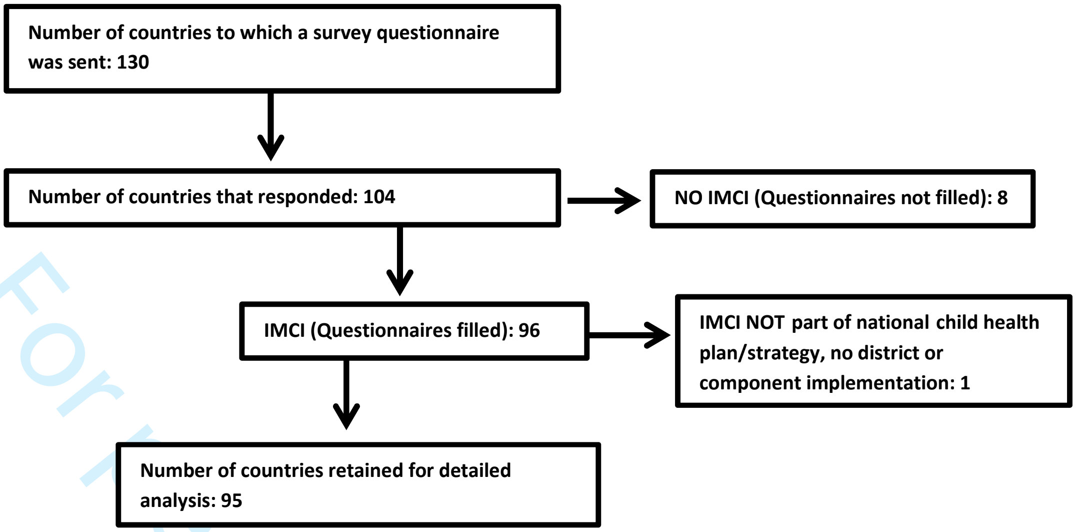

For peer review only

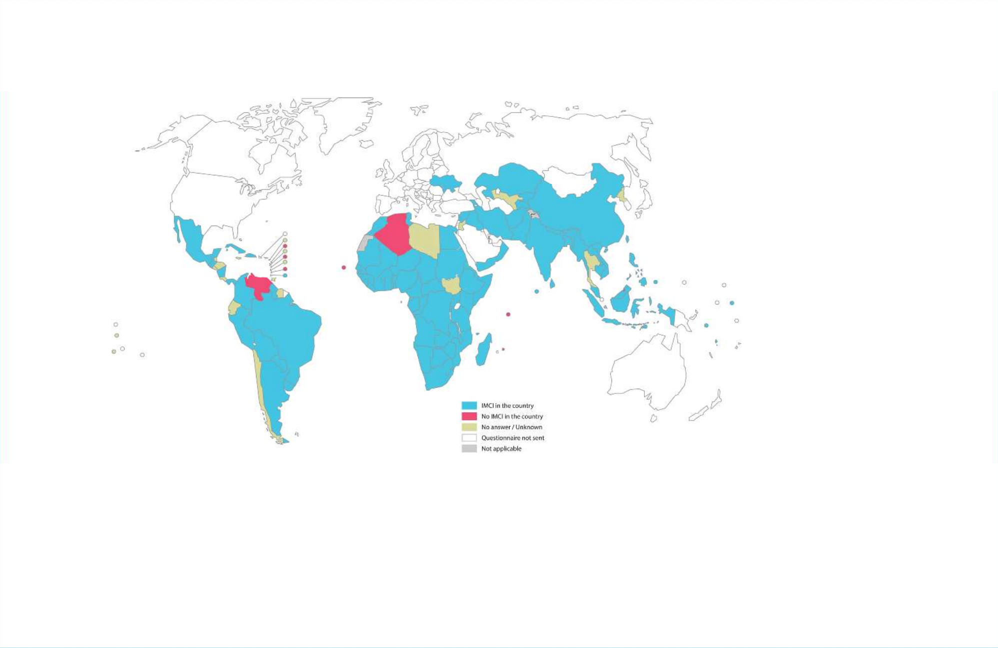

Figure 2: Survey summary map

279x182mm (300 x 300 DPI)

view only

Figure 3: Proportion of countries reporting government as primary funder of various IMCI expenditures at first level health facility by under-five mortality level

804x712mm (600 x 600 DPI)

For peer review only - http://bmjopen.bmj.com/site/about/guidelines.xhtml

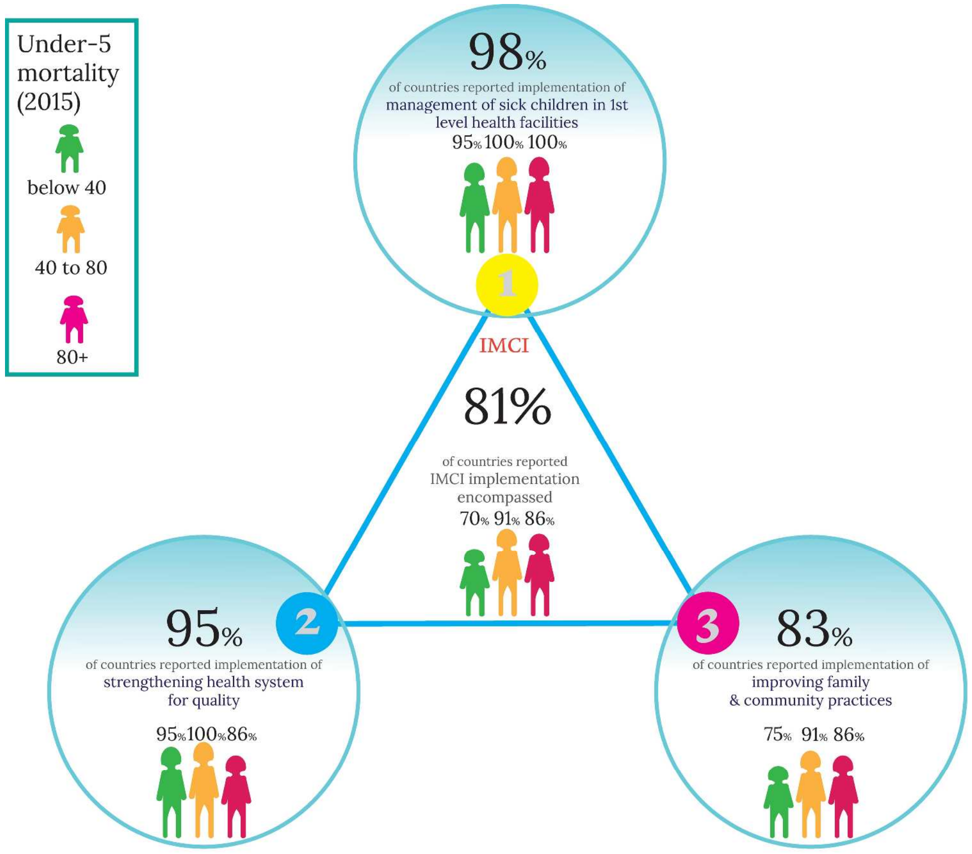

Figure 4. Implementation of the three IMCI components

336x418mm (300 x 300 DPI)

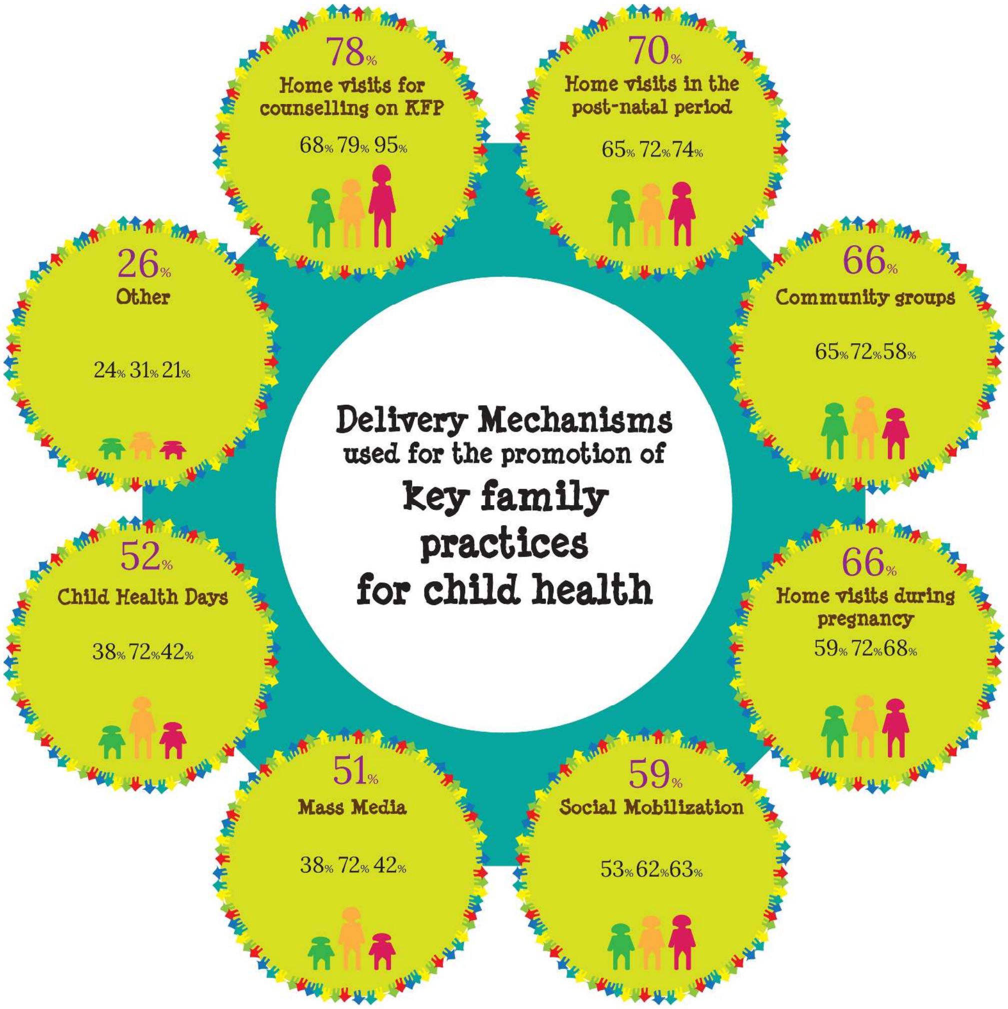

Figure 5. Main delivery mechanisms used for the promotion of key family practices related to child health

271x271mm (300 x 300 DPI)

STROBE Statement—checklist of items that should be included in reports of observational studies

	Item No	Recommendation	Action
**Title and abstract**	1	(*a*) Indicate the study's design with a commonly used term in the title or the abstract	Yes (Title; Page 1)
		(*b*) Provide in the abstract an informative and balanced summary of what was done and what was found	Yes (Pages 1 and 2)
**Introduction**			
Background/rationale	2	Explain the scientific background and rationale for the investigation being reported	Yes (Page 3)
Objectives	3	State specific objectives, including any prespecified hypotheses	Yes (Introduction, Page 3)
**Methods**			
Study design	4	Present key elements of study design early in the paper	Yes (First sentence of Methods, Page 3)
Setting	5	Describe the setting, locations, and relevant dates, including periods of recruitment, exposure, follow-up, and data collection	Yes (within context of this survey with countries as units of analysis – no recruitment or individual level analysis)
Participants	6	(*a*) *Cohort study*—Give the eligibility criteria, and the sources and methods of selection of participants. Describe methods of follow-up<br>*Case-control study*—Give the eligibility criteria, and the sources and methods of case ascertainment and control selection. Give the rationale for the choice of cases and controls<br>*Cross-sectional study*—Give the eligibility criteria, and the sources and methods of selection of participants	Not applicable - Human subjects not directly involved in the study
		(*b*) *Cohort study*—For matched studies, give matching criteria and number of exposed and unexposed<br>*Case-control study*—For matched studies, give matching criteria and the number of controls per case	Same as above
Variables	7	Clearly define all outcomes, exposures, predictors, potential confounders, and effect modifiers. Give diagnostic criteria, if applicable	Not applicable as this was a cross-sectional survey
Data sources/ measurement	8*	For each variable of interest, give sources of data and details of methods of assessment (measurement). Describe comparability of assessment methods if there is more than one group	Yes (Methods, Page 3 containing all detailed sources of data)
Bias	9	Describe any efforts to address potential sources of bias	Yes within context of survey design (Discussion - paragraph on limitations; Page 9)
Study size	10	Explain how the study size was arrived at	Yes within context of a

For peer review only - http://bmjopen.bmj.com/site/about/guidelines.xhtml

			survey – no sample calculations (Methods; Page 3)
Quantitative variables	11	Explain how quantitative variables were handled in the analyses. If applicable, describe which groupings were chosen and why	Yes (Methods, Page 3)
Statistical methods	12	(*a*) Describe all statistical methods, including those used to control for confounding	Not applicable
		(*b*) Describe any methods used to examine subgroups and interactions	Not applicable
		(*c*) Explain how missing data were addressed	Not applicable
		(*d*) *Cohort study*—If applicable, explain how loss to follow-up was addressed<br>*Case-control study*—If applicable, explain how matching of cases and controls was addressed<br>*Cross-sectional study*—If applicable, describe analytical methods taking account of sampling strategy	Not applicable
		(*e*) Describe any sensitivity analyses	None performed
**Results**			
Participants	13*	(a) Report numbers of individuals at each stage of study—eg numbers potentially eligible, examined for eligibility, confirmed eligible, included in the study, completing follow-up, and analysed	Yes, in the context of countries as unit of analysis (Methods, Page 3)
		(b) Give reasons for non-participation at each stage	Yes, same as above
		(c) Consider use of a flow diagram	Yes, same as above
Descriptive data	14*	(a) Give characteristics of study participants (eg demographic, clinical, social) and information on exposures and potential confounders	Yes within context of a survey with countries as unit of analysis (Web Appendix; Table 1)
		(b) Indicate number of participants with missing data for each variable of interest	
		(c) *Cohort study*—Summarise follow-up time (eg, average and total amount)	Not applicable
Outcome data	15*	*Cohort study*—Report numbers of outcome events or summary measures over time	-
		Case-control study—Report numbers in each exposure category, or summary measures of exposure	-
		Cross-sectional study—Report numbers of outcome events or summary measures	Yes (Results; Pages 4-8)
Main results	16	(*a*) Give unadjusted estimates and, if applicable, confounder-adjusted estimates and their precision (eg, 95% confidence interval). Make clear which confounders were adjusted for and why they were included	Not applicable
		(*b*) Report category boundaries when continuous variables were categorized	Not applicable
		(*c*) If relevant, consider translating estimates of relative risk into absolute risk for a meaningful time period	Not relevant
Other analyses	17	Report other analyses done—eg analyses of subgroups and interactions, and sensitivity analyses	Qualitative analysis of open-ended questions

For peer review only - http://bmjopen.bmj.com/site/about/guidelines.xhtml

			(Results; Page 7)
**Discussion**			
Key results	18	Summarise key results with reference to study objectives	Yes (Discussion; Pages 8 and 9)
Limitations	19	Discuss limitations of the study, taking into account sources of potential bias or imprecision. Discuss both direction and magnitude of any potential bias	Yes (Page 9)
Interpretation	20	Give a cautious overall interpretation of results considering objectives, limitations, multiplicity of analyses, results from similar studies, and other relevant evidence	Yes (Page 9)
Generalisability	21	Discuss the generalisability (external validity) of the study results	Not applicable in the context of this study
**Other information**			
Funding	22	Give the source of funding and the role of the funders for the present study and, if applicable, for the original study on which the present article is based	Yes (Page 10)

*Give information separately for cases and controls in case-control studies and, if applicable, for exposed and unexposed groups in cohort and cross-sectional studies.

Note: An Explanation and Elaboration article discusses each checklist item and gives methodological background and published examples of transparent reporting. The STROBE checklist is best used in conjunction with this article (freely available on the Web sites of PLoS Medicine at http://www.plosmedicine.org/, Annals of Internal Medicine at http://www.annals.org/, and Epidemiology at http://www.epidem.com/). Information on the STROBE Initiative is available at www.strobe-statement.org.

For peer review only

Global implementation survey of the Integrated Management of Childhood Illness (IMCI) – twenty years on

Journal:	*BMJ Open*
Manuscript ID	bmjopen-2017-019079.R1
Article Type:	Research
Date Submitted by the Author:	17-Oct-2017

Complete List of Authors:	Boschi-Pinto, Cynthia; WHO, Maternal, Newborn, Child and Adolescent Health; Universidade Federal Fluminense, Instituto de Saúde Coletiva<br>Labadie, Guilhem; WHO, Maternal, Newborn, Child and Adolescent Health<br>Dilip, Thandassery; World Health Organization, Maternal, Newborn, Child and Adolescent Health;<br>Oliphant, Nicholas; The Global Fund to Fight AIDS, Tuberculosis, and Malaria (Geneva, Switzerland), Public Health Monitoring and Evaluation ; UNICEF, NY<br>Dalglish, Sarah; World Health Organization, Maternal, Newborn, Child and Adolescent Health<br>Aboubaker, Samira; World Health Organization, Maternal, Newborn, Child and Adolescent Health<br>AGBODJAN-PRINCE, Olga; World Health Organization, Regional Office for Africa<br>Desta, Teshome; World Health Organization, Inter-country Support Team for East and Southern Africa<br>Habimana, Phanuel; World Health Organization/Regional Office for Africa,<br>Butrón-Riveros, Betzabé ; World Health Organization, Regional Office for the Americas<br>Al-Raiby, Jamela; World Health Organization, Regional Office for Eastern Mediterranean<br>Siddeeg, Khalid; World Health Organization, Regional Office for the Eastern Mediterranean<br>Kuttumuratova, Aigul ; World Health Organization, Regional Office for Europe<br>Weber, Martin; World Health Organization, Regional Office for Europe<br>Mehta, Rajesh; WHO Regional Office for South-east Asia<br>Neena, Raina; WHO Regional Office for South-east Asia<br>DAELMANS, Bernadette; World Health Organization, Chils and Adolescent Health and Development<br>Diaz, Theresa; World Health Organization, Maternal, Newborn, Child and Adolescent Health; UNICEF, NY

**Primary Subject Heading**:	Global health
Secondary Subject Heading:	Health policy, Paediatrics, Public health
Keywords:	Child Health, IMCI, Strategic Review, IMCI Global Survey

For peer review only - http://bmjopen.bmj.com/site/about/guidelines.xhtml

For peer review only

Global implementation survey of the Integrated Management of Childhood Illness (IMCI) – twenty years on

Authors

Cynthia Boschi-Pinto[a,b], Guilhem Labadie[a], Thandassery Ramachandran Dilip[a], Nicholas Oliphant[c,d], Sarah Dalglish[a], Samira Aboubaker[a], Adjoa Agbodjan-Prince[e], Teshome Desta[f], Phanuel Habimana[g], Betzabe Butron-Riveros[h], Jamela Al-Raiby[i], Khalid Siddeeg[i], Aigul Kuttumuratova[j], Martin Weber[j], Rajesh Mehta[k], Neena Raina[k], Bernadette Daelmans[a], Theresa Diaz[a]

[a] Department of Maternal Newborn Child and Adolescet Heath, World Health Organization
[b] Instituto de Saúde Coletiva, Universidade Federal Fluminense
[c] The Global Fund to Fight AIDS, Tuberculosis, and Malaria (current)
[d] UNICEF during the development of the survey (previous)
[e] World Health Organization, Regional Office for Africa, Ouagadougou, Burkina Faso
[f] World Health Organization, Regional Office for East and Southern Africa, Harare, Zimbabwe
[g] World Health Organization, Regional Office for Africa, Brazzaville, Congo
[h] World Health Organization, Regional Office for the Americas
[i] World Health Organization, Regional Office for Eastern Mediterranean
[j] World Health Organization, Regional Office for Europe
[k] World Health Organization, Regional Office for South-east Asia

Corresponding author: Cynthia Boschi-Pinto
World Health Organization, department of Maternal Newborn Child and Adolescent Health
Avenue Appia 20, 1211 Geneva, Switzerland
Email address: pintoc@who.int
Phone number: +41227913453
Fax number: +41227914853

Abstract

Objective: To assess the extent to which Integrated Management of Childhood Illnesses (IMCI) has been adopted and scaled up in countries.

Setting: The 95 countries that participated in the survey are home to 82% of the global under-five population and account for 95% of the 5.9 million deaths that occurred among children less than five years of age in 2015; 93 of them are LMICs.

Methods: We conducted a cross-sectional self-administered survey. Questionnaires and data analysis focused on providing: 1) a general overview of the current level of organization and financing of IMCI at

For peer review only - http://bmjopen.bmj.com/site/about/guidelines.xhtml

country level; 2) a description of implementation of IMCI's three original components; and 3) reported innovations, barriers and opportunities in expanding access to care for the sick child. A single data file was created using all information collected. Analysis was performed using STATA version 11.

Participants: In-country teams consisting of representatives of the ministry of health (MoH) and country offices of the World Health Organization (WHO) and United Nations Emergency Fund for Children (UNICEF).

Results: Eighty-one percent of countries reported that IMCI implementation encompassed all three components. Almost half (46%; 44 countries) reported implementation in 90% or more districts as well as all three components in place (full implementation). These full implementer countries were 3.6 [95% CI 1.5 8.9] times more likely to achieve MDG4than other (not full implementer) countries. Despite these high reported implementation rates, the strategy is not reaching the children who need it most as implementation is lowest in high mortality countries (39%; 7/18), where it is probably most needed.

Conclusion: This survey is a unique opportunity to better understand how and in which direction the implementation of IMCI has evolved over 20 years since its inception. Results obtained are extremely informative and should be used to assist in formulating strategies, policies and activities to support improvements in health and survival of children and to help achieving the UN health-related, post-2015 SDGs.

Article Summary

Strengths and limitations of this study

This survey is a unique opportunity to better understand how and in which direction the implementation of IMCI has evolved over 20 years since its inception and to help steer future child health directions

Limitations are mostly due to the study design: respondents may not feel comfortable providing answers that present the country in an unfavourable manner and therefore may not provide accurate answers

The WHO African region is likely to be overrepresented in the survey responses, but disaggregation by mortality level and income groups reduces the effect of this overrepresentation on the interpretation of results

For peer review only - http://bmjopen.bmj.com/site/about/guidelines.xhtml

Introduction

WHO and UNICEF introduced Integrated Management of Childhood Illness (IMCI) in the mid-1990s as a strategy to improve child survival in countries with more than 40 deaths per 1000 live births, and provide integrated prevention, treatment and care for the sick child (1). The strategy was later expanded to include care for sick newborns under one week of age, and has been regularly updated to reflect advancements in technical knowledge. Over 100 countries have adopted IMCI and implemented, in whole or in part, its three components: 1) improving health worker skills, 2) strengthening health systems, and 3) improving family and community practices. Evidence suggests that IMCI has contributed to reductions in child mortality over the MDG era, and a recent Cochrane review found the strategy was associated with a 15% reduction in child mortality when activities were implemented in health facilities and communities (2).

Initial reviews of IMCI implementation, notably the 2003-05 Multi-Country Evaluation (3) and the 2003 Analytic Review (4), used in-depth looks at specific country contexts to draw broader lessons about implementation dynamics. WHO has also regularly surveyed countries about their adoption and implementation of the IMCI strategy, and has occasionally published maps on its website showing the extent of implementation. However, it has not provided a comprehensive description of global implementation worldwide, with details on country variations, implementation barriers and facilitators, local innovations, and other particulars. Meanwhile UNICEF has run a number of surveys about countries' adoption and implementation of integrated Community Case Management (iCCM), a strategy similar to IMCI focusing on provision of care for sick children by community health workers (CHWs), however the data produced focused mainly on implementation in sub-Saharan African countries (5).

As part of the Strategic Review of the Integrated Management of Childhood Illnesses (IMCI) (6,7), a global survey was carried out in April – June 2016 to assess the extent to which this strategy has been adopted and scaled up in countries over the past two decades. This article reports on its main results and provides an overview of the current status of global implementation of IMCI as informed by countries. The article also presents the main strengths and barriers in IMCI implementation, as stated by respondents, as well as suggested ways forward.

Methods

This was a cross sectional self-administered global survey carried out from April 2016 to June 2016. The survey instrument [Web Annex 1] was developed by WHO with inputs from UNICEF and was tested in three different WHO regions to ensure questions were clear and appropriate and to estimate the time needed to complete the questionnaire. The final version was made available in all six UN languages (Arabic, Chinese, English, French, Spanish and Russian). Questionnaires included mostly closed-ended questions. Countries identified the principal strengths of IMCI and barriers to its implementation by agreeing or disagreeing with a suggested list, and answered open-ended questions about future actions to sustain and improve implementation. Questions and data analysis focused on variables providing: 1) a general overview of the current level of organization and financing of IMCI at country level; 2) a description of implementation of IMCI's three original components and of some additional components, such as iCCM; and 3) the main innovations, barriers and opportunities used in expanding access to care for the sick child.

These structured questionnaires were sent to a total of 130 WHO Member States by the six WHO Regional Offices (ROs) that implemented the survey through the WHO country offices (COs), provided support and follow-up. ROs had different approaches when sending out questionnaires to countries, reflecting specificities from each region. In the African region, questionnaires were sent to all 47 WHO

For peer review only - http://bmjopen.bmj.com/site/about/guidelines.xhtml

member states. Four countries (Algeria, Cape Verde, Mauritius, and Seychelles) responded that they had not adopted IMCI and therefore did not participate in the survey. Responsible officers from South Sudan and São Tomé & Principe did not respond to the survey. In the WHO Region of the Americas, questionnaires were sent to all 33 low- and middle-income countries (LMICs) in the region, of which 19 (58%) responded. Four countries (Antigua & Barbuda, St Lucia, St Vincent & Grenadines and Venezuela) responded that they had not adopted IMCI and therefore 15 countries were included in the survey. Questionnaires were sent to 16 WHO countries in the Eastern Mediterranean region, known to have adopted and implemented IMCI. Responses were received from 14 countries. Of the 53 countries in the WHO European region, 15 have introduced and/or implemented IMCI at different points in time and on varying scale. Survey questionnaires were sent to seven WHO member states where WHO National Professional Officers (NPOs) were present, and six responded (86%). In the South-East Asia region, questionnaires were sent to all 11 WHO member states, of which 9 (82%) responded. Finally, questionnaires were sent to 16 low- and middle-income countries in the Western Pacific region, and 10 (69%) were included in the final detailed analysis. Respondents comprised in-country teams consisting of a combination of representatives of the ministry of health (MoH) and country offices of WHO and UNICEF or in some circumstance just representatives of the MoH. Respondents were IMCI experts with country experience. Responses were tracked by WHO regional offices to maximize return and clarify inconsistencies or omissions. Filled in questionnaires were then returned from countries to WHO Headquarters.

Data from excel questionnaires were initially converted into an excel database. The responses were checked for skip pattern and missing data were recoded. Clarification was sought from countries with missing data, of which some responded. Analyses included descriptive statistics that synthesize and organize the information obtained using numerical procedures (simple frequency and means in univariate and bivariate tables), and/or graphic techniques to describe the characteristics of IMCI in the respondent countries. Data analysis was disaggregated by levels of under-five mortality rates (U5MRs), gross national income (GNI) per capita, and extent of IMCI implementation. Countries were categorized into low (<40 deaths per 1000 live births), middle (40-79 deaths per 1000 live births) and high mortality (80+ deaths per 1000 live births). Countries were also classified by income level (low, middle, and high) according to current GNI per capita (2016) using World Bank data (8), and as high (>90% of districts reported to have implemented IMCI), middle (50-90% of districts reported to have implemented IMCI) and low implementers (<50% of districts reported to have implemented IMCI), according to the proportion of districts reported to have implemented the strategy. Information on under-five mortality (numbers and rates) for 2015 was obtained from the United Nations Inter-agency Group for Child Mortality Estimation (UN-IGME) report on levels and trends in child mortality (9). Information on under-five population for the year 2015 was obtained from the UN Population Division (10). A single data file was created containing all information collected. Responses to pre-coded questions are presented in this paper. Analysis was performed using STATA version 11.0 software. More detailed methods are described elsewhere (11).

As is common with self-administered questionnaires respondents may have inaccurately reported the level and extent of IMCI implementation, most likely reporting a more “positive” scenario than the reality.

Patient Involvement

This is a self-administered key-informant survey carried out at national level and no individual patient data was included.

For peer review only - http://bmjopen.bmj.com/site/about/guidelines.xhtml

Results

Participants and Descriptive Data

Questionnaires were sent to a total of 130 WHO Member States with a response rate of 80% [Web Table 1]. Of the 104 countries that responded to the survey, eight reported no IMCI in the country, leaving 96 countries for the general analysis. One country was dropped from the analysis because IMCI was not reported to be part of their national child health plan or strategy, no district was reported to be implementing IMCI and none of the three components was reported to be in place (Flow diagram).Of the 95 countries retained for final analysis (Figure 1), only two (Oman and Uruguay) are classified as high-income countries according to the World Bank; 64 are classified as middle-income and 29 as low-income. These 95 countries are home to 82% of the global under-five population and account for 95% of the 5.9 million deaths that occurred among children less than five years of age in 2015; 93 of them are LMICs.

The instruction for questionnaires to be filled in by a three in-country team was followed in only five countries. In the majority of countries only the MoH provided answers [Web Table 2]. For three countries, the information on respondents is not available.

Eighty-nine per cent (82/94) of countries had an IMCI focal point at national level, whereas only 68% (62/91) had one at the sub-national (regional or district) level. Among high mortality countries, 88% (17/20) had a national focal point, but less than half this proportion (42%; 8/19) had subnational focal points. The presence of regional/district focal point was much higher in countries with U5MRs below 80 per 1000 live births (75%; 54/72) than in countries with higher rates. The government is generally reported to be the principal funding source for IMCI expenditures at first level health facilities and in the community, except for the training courses and per diem. For the latter, donors constituted the main sources of funding. In Figure 2 we show the proportion of countries reporting government as primary funder of different elements of IMCI in first level health facility by under-five mortality level.

Main Results

Implementation of IMCI and its three components

For the purpose of the survey, IMCI is said to be implemented if it is included in national and district work plans and budgets, if district health facilities use the strategy to provide care for the sick child (with or without community-based IMCI), and if providers are trained in IMCI to care for the sick child at national and district levels. Of the 95 countries included in the full analysis, 81 responded to the question on the proportion of districts implementing IMCI. Of these 81 responding countries, 51 (63%) reported implementing IMCI in all districts

Figure 3 shows the proportion of countries reporting the components that had been encompassed during IMCI implementation. While 92 (98%) of the 94 responding countries reported having implemented the first component of IMCI (improving health worker skills), 89 (95%) reported implementing the second (strengthening health systems), and 78 (83%) responded having implemented the third (improving family and community practices). Overall, 76 (81%) of the 94 countries reported implementing all three components.

Forty-four countries have reported implementing IMCI in more than 90% of districts *and* also having all three IMCI components in place; these are considered full implementer countries. These countries are home to 160 million of the global under-five children.

For peer review only - http://bmjopen.bmj.com/site/about/guidelines.xhtml

First component – improving health worker skills

The original training course for health workers lasted 11 days, with 30% of time spent in clinical sessions (12). Most responding countries (42%; 39/92) used shortened or abridged versions (ranging from 6 to 8 days) of the original 11-daz course. Clinical practice was at least 25% of the training time in 79% of the countries (72/91). Nearly three quarters of countries (72%; 67/93) reported implementing some sort of IMCI pre-service training.

Generic case management guidelines and related training materials were targeted to be appropriate in the majority of LMICs where U5MR is higher than 40 per 1000 live births, and where there is transmission of *P. falciparum* malaria (13). Over the years, IMCI chart booklets have been continuously adapted to the context and changing scenarios of each country. Since the last global update in 2014, 81% (69/85) of countries reported having updated their national chart booklets. Recognizing the increased importance of the burden of newborn mortality, nearly all countries (95%; 89/94) have adapted IMCI guidelines to include care of the sick newborn in the first week of life. Development or revision of national treatment guidelines based on the 2013 revision of the WHO Pocket Book of Hospital Care for Children and its adaptation in referral facilities were reported by 55% (48/87) and 44% (39/88) of responding countries, respectively. Considering Emergency Triage Assessment and Treatment (ETAT), 23% of the 65 responding countries reported introducing them in at least 75% of hospitals. This proportion is remarkably lower (17%) in the 41 middle and high mortality countries than in the 24 low mortality countries (38%).

The most common conditions added to the guidelines in national adaptations were jaundice in newborn babies and young infants (77%; 72/93), HIV (63%; 59/93), sore throat (59%; 55/93), and skin conditions (44%; 41/93). Other illnesses frequently included are tuberculosis and dengue haemorrhagic fever. Adaptations varied according to regional or national epidemiological profile.

Second component – strengthening health systems

Improving and sustaining the quality of paediatric care is an integral part of strengthening health systems. An assessment of quality of paediatric hospital care was conducted in 42% (39/92) of responding countries. Fifty-two per cent (34/65) of middle and high implementer countries reported having such assessment compared to only 8% (1/13) of low implementer countries. Fifty-eight per cent of the 92 responding countries reported having a paediatric QoC improvement programme for health facilities in their MoH. Countries that report improvement programmes account together for nearly 3 million (50%) of all 5.9 million under-five deaths that occurred worldwide in 2015.

Supervision and monitoring are key factors for sustainable QoC and for strengthening the health system. Few countries (15%; 10/66) reported more than 75% of first level facilities with at least one supervisory visit in the last six months; half (33/66) had less than one quarter of these facilities receiving such visits. In general, monitoring of IMCI implementation has been carried out infrequently. Only 33% of countries (30/91) reported having a comprehensive IMCI monitoring and evaluation plan. Fifteen per cent (3/20) of high mortality countries reported having such a plan, while this proportion was 38% (27/71) in middle and low mortality countries. Still, more than two thirds of countries (70%; 66/94) reported that their Health Management Information System (HMIS) includes monitoring indicators for IMCI. Most of these indicators are collected at the primary health care level (83%; 54/65); only four countries reported having monitoring indicators for IMCI collected at community level.

In 2009, WHO launched the "Managing Programmes to Improve Child Health" course, which has been introduced in almost half (48%; 42/88) of responding countries. The number of such trained managers per under-five population varies widely, ranging from 1 manager per 840 thousand under-five children in Lao

For peer review only - http://bmjopen.bmj.com/site/about/guidelines.xhtml

PDR to 1 manager for 1500 children in Kiribati – a more than 500-fold difference. Tools for bottleneck analysis and strategic planning have been introduced in 33 of the 79 responding countries (42%).

The main innovations reported by countries are related to information communication technologies (ICTs), which include eHealth, mHealth and Rapid SMS. Use of such innovations in the implementation of IMCI was reported over six categories: data collection and reporting (70%; 60/86); supply chain management (58%; 51/88); behaviour change communication (54%; 48/89); health provider training and education (54%; 46/85); electronic decision support (49%; 42/85); and financial transactions and incentives (41%; 35/86).

Third component - Improving family and community practices

Improving family and community practices was reported as part of IMCI implementation by 83% (78/94) of responding countries (Figure 3). Eighty-nine per cent (48/54) of medium and high mortality countries report implementation of this component, while 75% (30/40) of low mortality countries reported implementing it. In general, activities to *promote key family practices for child health* have been implemented through home visits, and through social mobilization or community groups. The main delivery mechanisms used are illustrated in Figure 4. The majority of responding countries (71%; 45/63) reported that one community health worker (CHW) covered up to 1000 under-five population. Supervision for CHWs was mainly provided by IMCI trained supervisor (63%; 58/92) and through mentorship in health facilities (45%; 41/92).

Community care for children under five years of age was reported to be provided by more than three quarters (78%, 72/92) of responding countries. The proportion of countries with CHWs providing treatment for children, in general, was higher in high (85, 17/20) and medium (94%, 31/33) mortality countries as compared to low mortality countries (62%, 24/39). These variations are similar to those observed for the presence of community case management (CCM) policy in countries. Among the 72 countries that reported that CHWs provide care for children, 93% (67 countries) reported having CCM for diarrhoea. Of these, 80% (53/66) reported having the relevant written policy in place (one country did not respond). Conversely, six countries reported having the policy in place but not implementing it. Among the 72 countries where CHWs provided treatment for children, only 74% (53/72) reported that CHWs provide CCM for pneumonia. From these 53 countries, 43 (81%) reported having the relevant written policy in place. In 2012, the key elements of CCM were brought together in a package - integrated community case management (iCCM) (14), typically delivered by CHWs at the community level. It encompasses treatment for (i) childhood pneumonia with antibiotics, (ii) diarrhoea with zinc and oral rehydration salts and (iii) malaria with artemisinin combination therapy. Provision of iCCM was reported by 74% (53/72) of these responding countries.

Other aspects of community care, also supported by WHO and UNICEF, include the management of severe acute malnutrition (SAM) (15) and home visits (16). CCM for SAM was mentioned by 71% (51/72) of countries. Of the 95 countries participating in the survey, 65 reported that CHWs provide care for newborn babies. Of these countries, 91% (59/65) reported providing home visits to newborns. In comparison, 76% (55/72) provided home visits for children beyond the newborn period.

Strengths, Barriers, and Ways forward

The major strengths of IMCI most frequently identified by 93 responding countries were: the holistic approach to the child (90%; 84 countries), the rational use of medicine (89%; 83 countries), the quality of health services (87%; 81 countries), and the efficiency of service provision (80%; 74 countries).

For peer review only - http://bmjopen.bmj.com/site/about/guidelines.xhtml

At national level, the reported major barriers to implementing IMCI were budget for training (85%; 78/92), mentorship and supervision (74%; 68/92), cost or sustainability of activities (63%; 58/92), and availability of dedicated budget line (60%; 55/92). Major barriers reported at regional/district level were staff turnover (84%; 76/91), budget for training (82%; 75/91), and mentorship and supervision (74%; 67/91). Barriers reported at facility level were staff retention (80%; 73/91), mentorship and supervision (79%; 72/91), and staff motivation (74%; 67/91). High mortality and/or low income countries were more likely to report barriers related to budget for training, and medicine procurement and supply chain at national level. High implementer countries were more likely to report issues around mentorship and supervision, and less likely to report barriers on programme management and on political support and ownership.

When asked to suggest ways to strengthen child health services and make IMCI more relevant to today's needs, countries commonly advocated for better integration of current strategies into general health services, with a costed work plan and committed budget, as well as improved training at all levels, including pre-service training, and supportive supervision (helping to make things work, rather than checking to see if something went wrong) and mentoring. Health systems issues were said to limit implementation, with a strong need for a functioning supply chain, HMIS and monitoring and evaluation system. Better use of communications technologies was also suggested. Furthermore, compliance with national guidelines and good implementation practices should be recognized and rewarded. Community-level activities including outreach and home visits need more emphasis, as do the management of sick newborns at all levels. At policy level, strategic planning, improved internal coordination (via a dedicated focal person for child health, regular review meetings, national steering committee) and effective coordination with partners to overcome fragmentation would be essential. To achieve these goals, high-level advocacy and political support is said to be required, ensuring government leadership and ownership.

Conclusion

This article summarizes the information obtained from the first survey assessment of the status of IMCI implementation in 95 responding countries worldwide, twenty years after its launching in the mid-1990s (11). In 1998, 12 countries had already moved into a phase of expansion of IMCI's first component and had begun introducing the strategy's other two components (17). Twenty years later, coverage of IMCI is reported to be comprehensive in many target countries. However, although many countries have expanded aspects of implementation to a large proportion of their districts, few have achieved full scale up and, in many aspects, implementation remains incomplete. Moreover, regardless of the high reported implementation rates, the strategy is still not reaching the children who need it most - coverage of IMCI is lowest in high mortality countries. Nevertheless, it is worth noting that high implementer countries (≥90% of districts) with all three IMCI components in place are home for 160 million of the global under-five children. These full implementer countries were 3.6 [95% CI 1.5 – 8.9] times more likely to achieve MDG4 than other (not full implementer) countries. Also of note is the fact that among the 24 low and lower-middle income countries that have achieved MDG's 4 target, 18 (75%) are part of this group of 44 full implementer countries. Our results reinforce the original concept that full implementation of IMCI can lead to substantial impact on child health and survival

The training course for health workers developed by WHO and UNICEF is one of the key elements of IMCI strategy. Some limited evidence suggests the original 11-day training course to be more effective than shortened training (18); however, concerns regarding the costs and the absence of health workers from the services have been raised (19). Most countries reported using shortened or abridged versions of the original course. Flexibility to add conditions according to regional and national epidemiologic profile has facilitated adaptation of IMCI guidelines to a changing epidemiological profile. Supervision is the

For peer review only - http://bmjopen.bmj.com/site/about/guidelines.xhtml

weakest area under the second IMCI component, with only 15% of countries reporting more than three-quarters of first level facilities with at least one supervisory visit in the 6 months before the survey. The most commonly mentioned barriers have their roots in insufficient funding and weak health systems. Still, the major strengths identified and suggested ways forward offer important instruments to those thinking on and re-designing the future of child health and development.

There is widespread recognition that IMCI will only result in improvements in child health and survival if training activities are accompanied by effective efforts to strengthen health systems and reach children and mothers in the community. It has also been recognized that “strengthening health systems is the best way to safeguard against health crises” (20). Our findings show that low income countries particularly hailed IMCI efficiency in programming and in service provision, as well as equity in access and coverage of intervention. Sadly, results highlight the weakness of the implementation of IMCI second component. Supervision and mentorship were among the areas with the least impressive results. Especial attention should be directed to both the second component of IMCI and to the current status of implementation of IMCI in emergency settings.

This study has some limitations, most of which inherent to survey design. Firstly, respondents may not feel comfortable providing answers that present the country in an unfavourable manner and therefore may not provide accurate answers. Also, respondents may not always be fully aware of the situation in the country. To minimize this limitation, we have provided clear instructions for questionnaires to be responded by a three in-country team, but only five countries followed this instruction. Another 16 countries had questions answered by a two-respondent team. Bias due to non-response may also have occurred as respondents who chose to answer certain questions may differ from those who chose not to respond. Finally, the WHO African region is likely to be overrepresented in the survey responses, with 43% of the countries while the European region represents six per cent of them. Disaggregation by mortality level and income groups reduces the effect of this overrepresentation on the interpretation of results. Despite these limitations, this survey is a unique opportunity to better understand how and in which direction the implementation of IMCI has evolved over 20 years since its inception. It is also a unique opportunity to help steer future child health directions.

Despite the multiple and extensive constraints in IMCI implementation reported here, the findings of this survey provide an indication of the value and esteem that country-level stakeholders assign to IMCI. Results point to a unique opportunity to help steer future policies, programmes and strategies. Given the many competing priorities of survey respondents, the 80% response rate obtained reveals the interest IMCI still elicits, especially in low and middle income countries, and suggests a strengthened IMCI has a role to attend the call for “Survive, Thrive, Transform” from the Global Strategy for Women’s Children’s and Adolescents’ Health 2016-2030 (21). Truly, IMCI has generated a paradigm shift towards integrated programming for child health globally and in countries. This must be built upon in the current era of the sustainable development goals (SDGs), when global leaders have committed to reach a grand convergence in child health and end preventable newborn and child mortality. Full implementation of IMCI in health facilities and communities with a critical focus on health system strengthening and on emergency crises will therefore be decisive for countries to secure universal health care and to help achieve the UN health-related, post-2015 SDGs.

Ackowledgements

We thank our colleagues from WHO and UNICEF Country Offices and those from the Ministries of Health for their contribution on responding to the survey questionnaires and on providing follow-up of the survey.

For peer review only - http://bmjopen.bmj.com/site/about/guidelines.xhtml

Disclaimer

The authors* are staff members of the World Health Organization. The authors alone are responsible for the views expressed in this publication and they do not necessarily represent the decisions or policies of the World Health Organization.

Competing Interest

All authors have completed the Unified Competing Interest form (available on request from the corresponding author) and declare: no support from any organisation for the submitted work; no financial relationships with any organisations that might have an interest in the submitted work in the previous three years, no other relationships or activities that could appear to have influenced the submitted work.

Copyright

The Corresponding Author has the right to grant on behalf of all authors and does grant on behalf of all authors, an exclusive licence (or non exclusive for government employees) on a worldwide basis to the BMJ Publishing Group Ltd to permit this article to be published in BMJ editions and any other BMJPGL products and sublicences such use and exploit all subsidiary rights, as set out in our licence.

Funding

The Strategic Review of IMCI and iCCM was funded by the Bill and Melinda Gates Foundation; the authors donated their time to writing the article; the publication fees for the supplement were funded by the Health Systems Research Unit, South African Medical Research Council.

Contributors and sources

The authors bring a combination of expertise in epidemiology, demography, statistics, child health interventions and policies, public health, and advocacy. CBP conceptualized and wrote the first drafts of the paper. GL, TRD and NO performed the analysis. GL conceptualized and performed the illustrations and revised the first draft. SD analyzed the qualitative information and wrote the last session of the paper (Strengths, barriers and ways forward). BD and SA developed the questionnaire. SA revised several versions of the paper, providing advice on IMCI specific issues; helped coordinate the distribution and application of questionnaires and followed-up with WHO Regional Offices. AA-P, TD, PH, BB-R, JA-R, KS, AK, MW, RM, and NR organized, tested, followed-up and reviewed the questionnaires sent to countries. All authors contributed intellectual content and critical revisions to the manuscript. All authors approved the final manuscript. CBP is guarantor.

Data Sharing

All data will be available in public domain [IMCI Global Implementation Survey Report at: http://who.int/maternal_child_adolescent/documents/imci-global-survey-report/]

For peer review only - http://bmjopen.bmj.com/site/about/guidelines.xhtml

References

(1) WHO (1997). *First Global Review and Coordination Meeting on Integrated Management of Childhood Illness (IMCI).* [online] Santo Domingo: World Health Organization. Available at: http://apps.who.int/iris/bitstream/10665/64629/1/WHO_CHD_97.11.pdf

(2) Gera T, Shah D, Garner P, Richardson M, Sachdev HS. Integrated management of childhood illness (IMCI) strategy for children under five. Cochrane Database Syst Rev. 2016 Jun 22;(6):CD010123. doi: 10.1002/14651858.CD010123.pub2.

(3) Bryce J, Victora CG, Habicht JP, Black RE, Scherpbier RW; MCE-IMCI Technical Advisors. Programmatic pathways to child survival: results of a multi-country evaluation of Integrated Management of Childhood Illness. *Health Policy Plan*. 2005 Dec;20 Suppl 1:i5-i17.

(4) WHO (2003b). *The analytic review of the Integrated Management of Childhood Illness strategy Final report.* Available at: http://www.who.int/maternal_child_adolescent/documents/9241591730/en/ [Accessed 17 Jun. 2017].

(5) Rasanathan K, Muñiz M, Bakshi S, Kumar M, Solano A, Kariuki W, George A, Sylla M, Nefdt R, Young M, Diaz T. Community case management of childhood illness in sub–Saharan Africa – findings from a cross–sectional survey on policy and implementation. *J Glob Health* 2014 Dec; 4(2): 020401. doi: 10.7189/jogh.04.020401 PMCID: PMC4267096

(6) WHO. "Towards a Grand Convergence for child survival and health: A strategic review of options for the future building on lessons learnt from IMNCI." Geneva: WHO, 2016. Available at: http://www.who.int/maternal_child_adolescent/documents/strategic-review-child-health-imnci/en/

(7) Dalglish et al. Methods for the Strategic Review of IMNCI and iCCM programmes BMJ 2017 (paper 2 in the supplement – TBD)

(8) World Bank list of economies (December 2016) https://datahelpdesk.worldbank.org/knowledgebase/articles/906519-world-bank-country-and-lending-groups. Accessed on 26.01.2017

(9) WHO/ The World Bank/ United Nations Population Division. Levels & Trends in Child Mortality Report 2015. Estimates developed by the UN Inter-agency Group for Child Mortality Estimates. Available at: http://www.who.int/maternal_child_adolescent/documents/levels_trends_child_mortality_2015/en/

(10) United Nations, Department of Economic and Social Affairs, Population Division (2015). World Population Prospects: The 2015 Revision, custom data acquired via website: https://esa.un.org/unpd/wpp/Download/Standard/Population/

(11) WHO. IMCI Global Implementation Survey Report. Available at: http://who.int/maternal_child_adolescent/documents/imci-global-survey-report/

(12) WHO. IMCI Planning Guide. WHO, 1999. Available at: http://apps.who.int/iris/bitstream/10665/66720/1/WHO_CHS_CAH_99.1_eng.pdf

(13) WHO/UNICEF. IMCI Information Package, 1999. Available at: http://apps.who.int/iris/bitstream/10665/65002/4/WHO_CHS_CAH_98.1D_eng.pdf

(14) UNICEF (2012) Integrated Community Case Management: WHO/UNICEF Joint Statement. UNICEF, New York.

For peer review only - http://bmjopen.bmj.com/site/about/guidelines.xhtml

(15) WHO (2007), Community-based management of Severe Acute Malnutrition: Joint statement by WHO, WFP and UNICEF. Malnutrition: Joint statement by WHO, WFP and UNICEF. WHO, Geneva.

(16) WHO/Unicef (2009) WHO/Unicef Joint Statement on Home Visits for the Newborn Child: A strategy to Improve Survival. WHO, Geneva.

(17) Tulloch J. Integrated approach to child health in developing countries. Lancet 1999;354 Suppl 2;SII16-20

(18) Rowe AK, Rowe SY, Holloway KA et al. Does shortening the training on Integrated Management of Childhood Illness guidelines reduce its effectiveness? A systematic review. *Health Policy and Planning* 2012; 27:179-93.

(19) Goga AE, Muhe LM, Forsyth K, Chopra M, Aboubaker S, Martines J, Mason EM. Results of a multi-country exploratory survey of approaches and methods for IMCI case management training. *Health Res Policy Syst* 2009; 7-18.

(20) Ghebreyesus TA. All roads lead to universal health coverage. *Lancet Glob Health* 2017; Sep; 5(9): e839-e840.doi:10.1016/ S2214-109X(17)30295-4. Epub 2017 Jul 17.

(21) Global Strategy for Women's, Children's and Adolescents Health 2016-2030. Available at: http://www.who.int/life-course/partners/global-strategy/en/

List of Figures:

Figure 1: Survey summary map

Figure 2: Proportion of countries reporting government as primary funder of various elements of IMCI in first level health facility by under-five mortality level

Figure 3. Implementation of the three IMCI components

Figure 4. Main delivery mechanisms used for the promotion of key family practices related to child health

For peer review only - http://bmjopen.bmj.com/site/about/guidelines.xhtml

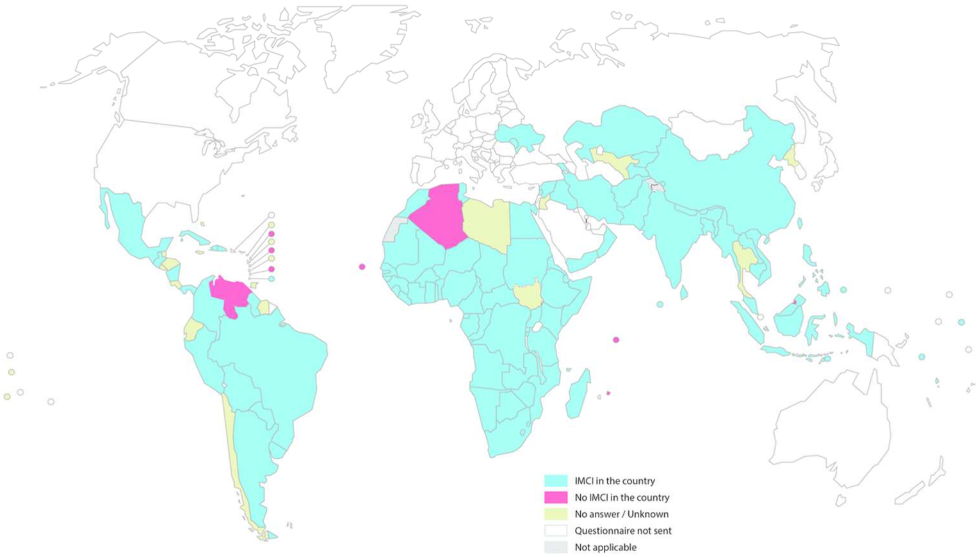

Figure 1: Survey summary map

120x69mm (300 x 300 DPI)

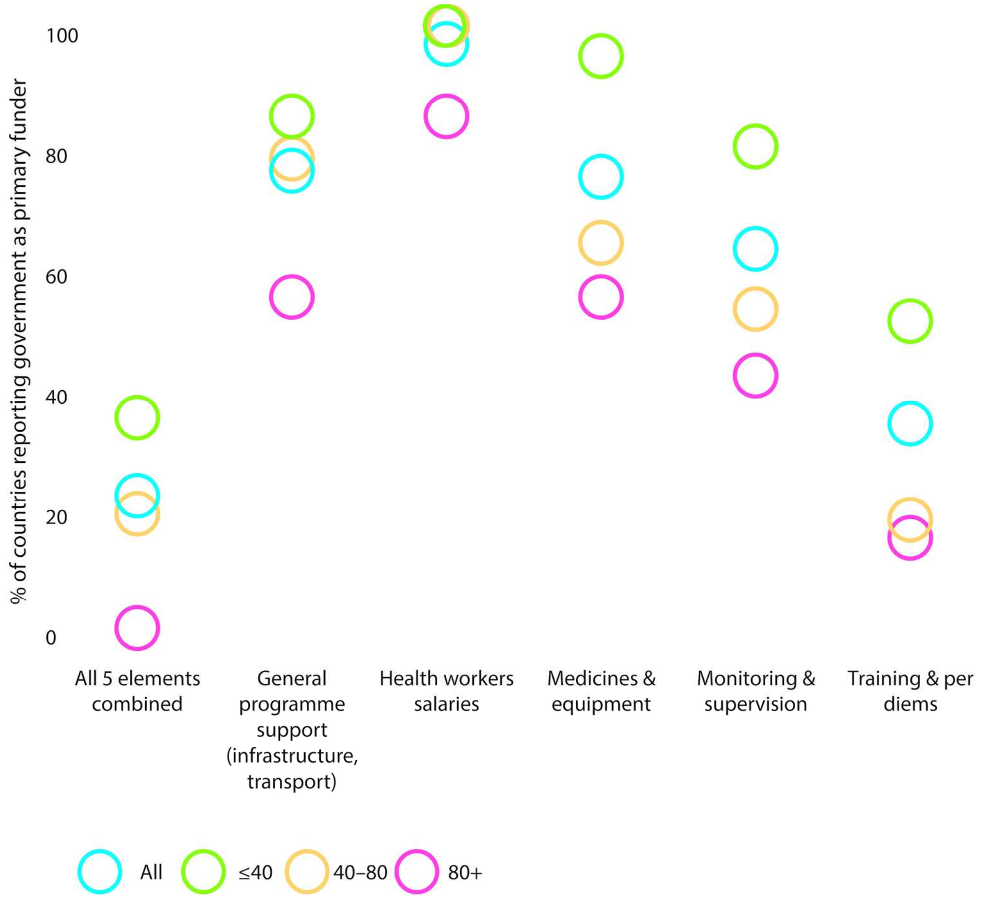

Figure 2: Proportion of countries reporting government as primary funder of various elements of IMCI in first level health facility by under-five mortality level

186x166mm (300 x 300 DPI)

nly

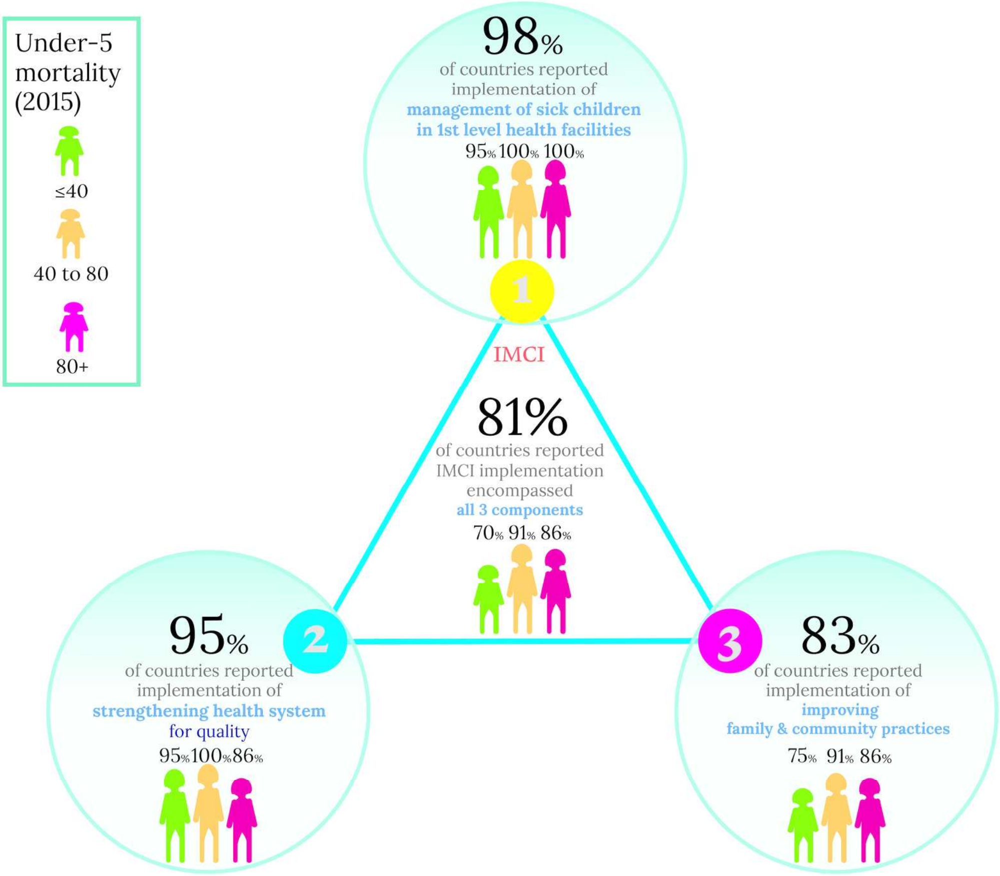

Figure 3. Implementation of the three IMCI components

209x185mm (300 x 300 DPI)

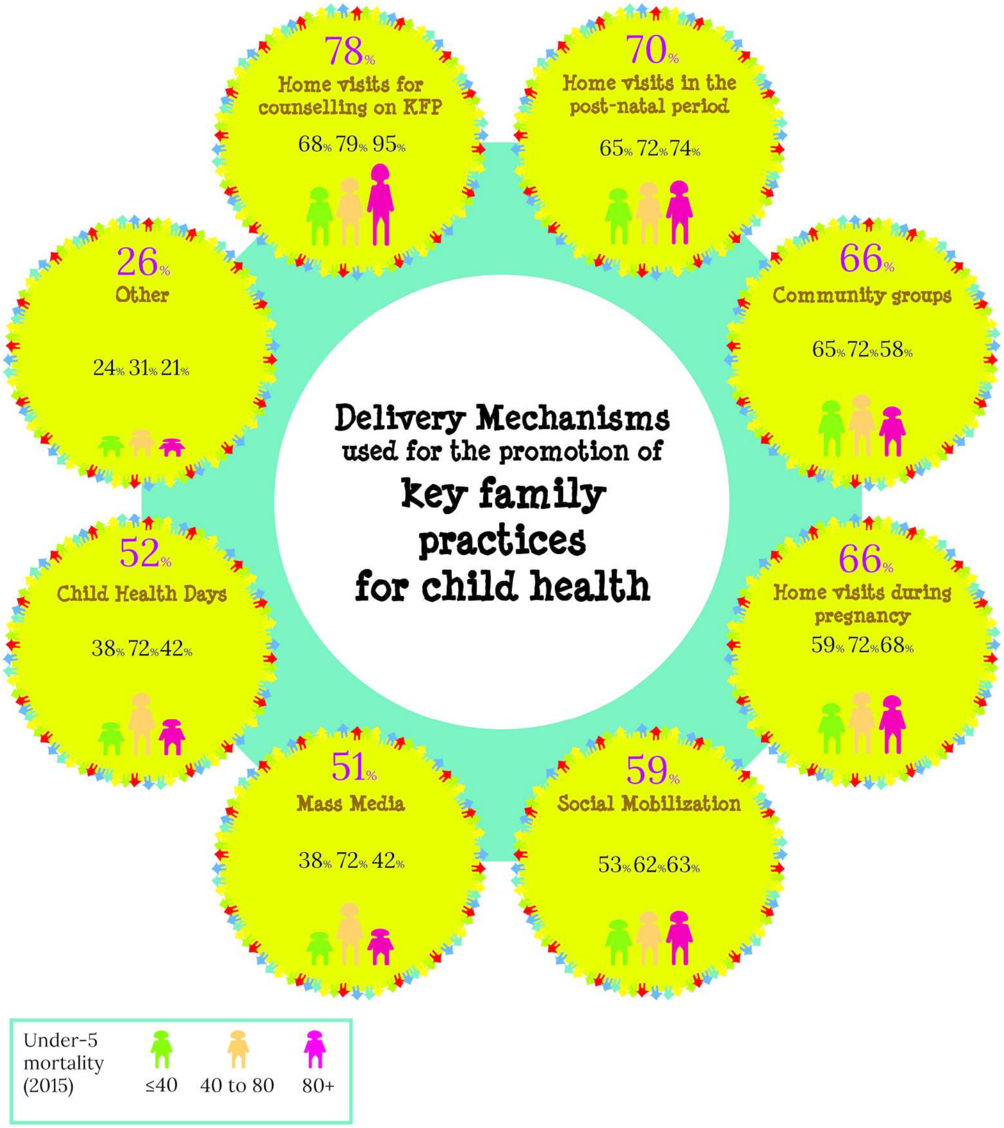

Figure 4. Main delivery mechanisms used for the promotion of key family practices related to child health

236x265mm (300 x 300 DPI)

Flow diagram

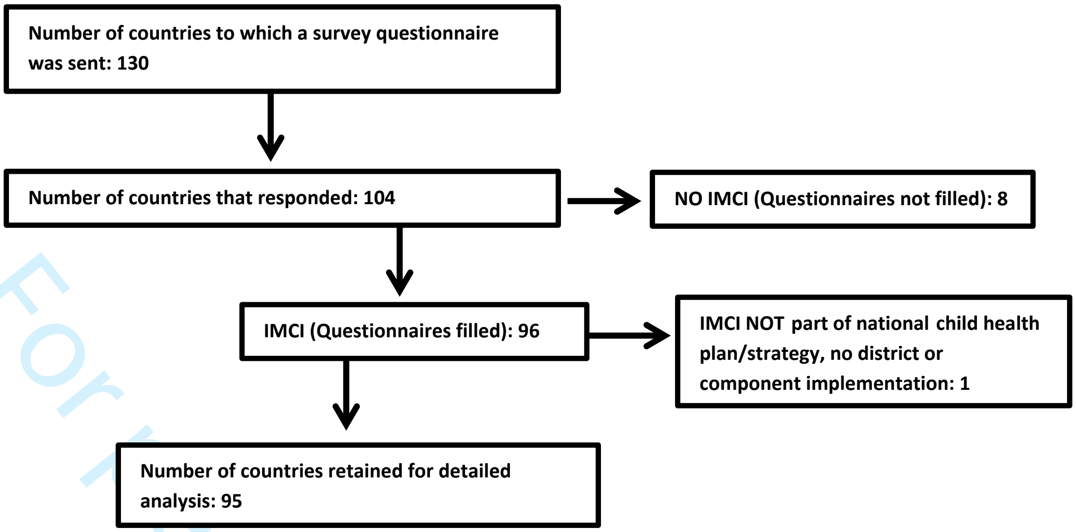

For peer review only

Web Appendix

Table 1. Response Rate to IMCI Global Implementation Survey

WHO Region	Total number of WHO MS	Member States (N) to which a survey questionnaire was sent	WHO MS that responded*	WHO MS that reported no IMCI (did not participate in the survey)	WHO MS that responded IMCI not in national plan/no district implementing nor component implemented	Response rate
**African**	47	47	45	4		95.7
**Americas**	35	33	19	4		57.6
**Eastern Mediterranean**	21	16	14			87.5
**Europe**	53	7	6			85.7
**South-east Asia**	11	11	9			81.8
**Western Pacific**	27	16	11		1	68.8
**Total**	**194**	**130**	**104**	**8**	**1**	80.0

MS = Member State; * Includes those MSs that responded not having adopted IMCI and therefore not answering the questions of the survey

Table 2. Respondents to IMCI Global Implementation Survey by WHO Region

WHO Region	Questionnaires included in final analysis	Three-team response (MoH/ WHO/ UNICEF)	Response by MoH/ WHO team	Response by MoH alone	Response by WHO alone	Response by WHO/ UNICEF team	Unknown
**African**	41	1	1	22	14	-	3
**Americas**	15	-	5	9	1	-	-
**Eastern Mediterranean**	14	3	7	4	-	-	-
**Europe**	6	1	-	3	1	1	-
**South-east Asia**	9	-	-	6	3	-	-
**Western Pacific**	10	-	1	9	-	-	-
**Total**	**95**	**5**	**15**	**52**	**19**	**1**	**3**

For peer review only

World Health Organization

IMCI Strategic Review: Survey on Global Implementation

Instructions:

After two decades of implementation, there is recognition that the **Integrated Management of Childhood Illness (IMCI) strategy requires review and repositioning**. WHO and partners are conducting a global assessment to provide **strategic directions on its recommendations for treating the sick child**, to contribute to the goal of ending preventable newborn and child mortality by 2030.

Responses from this survey will provide an **overview of global implementation** to assess gaps and help refine and **reposition IMCI at facility and community levels**. There are **4 worksheets** in this questionnaire, which we estimate will take 45 minutes to complete, ideally in a **team setting** including focal points for for IMCI and CCM in UNICEF and WHO Country Offices and the Ministry of Health.

To show our thanks for your effort, we are offering a **prize** to one randomly selected country per WHO region (6 prizes in total) for countries who complete and send the questionnaire by the required date (25 April 2016).

Return the completed questionnaire to your regional office with copy to Dilip THANDASSERY (thandasseryd@who.int) no later than **25th April 2016**.

Thank you for your collaboration!

Identification

1 Country

2 Lead respondent to this questionnaire

a Name

b Title / position

c Telephone

d Email

3 Date of completion

(day-month-year)

POLICY and GUIDELINES

Srl no	Question	Responding options	Mark answers below
101	Has the country developed/adopted a national child survival/ health strategy/plan to reduce child mortality?	Yes<br>No<br>Unknown	Select your answer
102	*If yes,*<br>What is the start date?<br>What is the end date?	Enter the year<br>Enter the year	
103	Is IMCI part of this national child health/survival strategy?	Yes<br>No<br>Unknown	Select your answer
104	If Yes,<br>In how many districts is IMCI currently implemented?	Enter the number of districts	
105	How many districts are there in the country?	Enter the number of districts	
106	What does IMCI implementation* encompass in your country?<br><br>*See below for definition of implementation	a. Improving management of sick children in first level health facilities<br>b. Treating sick children in the community<br>c. Improving management of sick children in referral facilities<br>d. Strengthening the health system for quality child care services<br>e. Improving family and community practices for child health	Check all that apply<br>☐<br>☐<br>☐<br>☐<br>☐
107	Are there any strategies other than IMCI used for the management of common childhood conditions? If yes, specify	Yes<br>No<br>Unknown	Select your answer
108	If *Yes* in Question 107, specify other strategies	______________________	
109	Has the national child health/survival strategy to reduce child mortality been costed?	Yes<br>No<br>Unknown	Select your answer
110	Is there a national health insurance scheme covering children under 5?	Yes<br>No<br>Unknown	Select your answer
111	If *Yes,*<br>Is provision of IMCI a criterion for reimbursement?	Yes<br>No<br>Unknown	Select your answer
112	Are consultations free for sick children under 5?	Yes<br>No<br>Unknown	Select your answer
113	Are medicines free for sick children under 5?	Yes<br>No<br>Unknown	Select your answer
NATIONAL IMCI GUIDELINES FOR FIRST LEVEL HEALTH FACILITIES			
114	Do IMCI guidelines include care of sick newborns in the first week of life?	Yes<br>No<br>Unknown	Select your answer
		a. Dengue	Check all that apply<br>☐

For peer review only

115	In addition to the generic IMCI guidelines, which conditions have been added in your national adaptation (Check all that apply):	b. Tuberculosis<br>c. HIV<br>d. Skin conditions<br>e. Sore throat<br>f. Jaundice<br>g. Other (Specify) ___________<br>h. Unknown/ not applicable	☐<br>☐<br>☐<br>☐<br>☐<br>☐<br>☐
116	When was the last update of the IMCI chart booklet completed?	Enter the year	
117	If in 2014, was the update based on the 2014 WHO Generic IMCI chart booklet?	Yes<br>No<br>Unknown	Select your answer
118	If yes, which conditions have been updated?	Cough or difficult breathing<br>Diarrhoea<br>Fever<br>HIV<br>Malnutrition<br>Anemia	Check all that apply<br>☐<br>☐<br>☐<br>☐<br>☐<br>☐
NATIONAL IMCI GUIDELINES FOR REFERRAL LEVEL HEALTH FACILITIES			
119	Has the country conducted an assessment of quality of care to improve management of childhood illnesses in referral hospitals?	Yes<br>No<br>Unknown	Select your answer
120	Has the country updated national treatment guidelines based on the 2013 edition of the WHO's Pocket book for hospital care for children?	Yes<br>No<br>Unknown	Select your answer
NATIONAL IMCI GUIDELINES FOR IMPROVING KEY FAMILY PRACTICES			
121	Are activities to promote key family practices for child health being implemented?	Yes<br>No<br>Unknown	Select your answer
122	If yes Q121, have counselling cards on key family practices been developed?	Yes<br>No<br>Unknown	Select your answer
123	If yes Q121, what are the delivery mechanisms used?	Home visits during pregnancy<br>Home visits in the postnatal period<br>Home visits for counselling on key family practices<br>Community groups<br>Child health days<br>Mass media<br>Social mobilization<br>Other	Check all that apply<br>☐<br>☐<br>☐<br>☐<br>☐<br>☐<br>☐<br>☐
NATIONAL GUIDELINES FOR CARING FOR NEWBORNS AND CHILDREN IN THE COMMUNITY			
124	Do community health workers (CHWs) provide care for newborns and babies in your country?	Yes<br>No<br>Unknown	Select your answer
		Community management of diarrhoea<br>Community management of pneumonia with antibiotics	Check all that apply<br>☐<br>☐

For peer review only

125	If yes in Q124, check all that apply	Community management of fever<br>Community management of severe malnutrition<br>Integrated community case management of diarrhoea, pneumonia, fever, and malnutrition<br>Home visits for newborn health in the postnatal period<br>Home visits for children beyond the newborn period<br>Not permitted	☐<br>☐<br>☐<br>☐<br>☐<br>☐
126	Do community health workers (CHWs) provide care for children in your country?	Yes<br>No<br>Unknown	Select your answer
127	If yes in Q126, check all that apply	Community management of diarrhoea<br>Community management of pneumonia with antibiotics<br>Community management of fever<br>Community management of severe malnutrition<br>Integrated community case management of diarrhoea, pneumonia, fever, and malnutrition<br>Home visits for newborn health in the postnatal period<br>Home visits for children beyond the newborn period<br>Not permitted	Check all that apply<br>☐<br>☐<br>☐<br>☐<br>☐<br>☐<br>☐<br>☐
128	Is there a written national policy for community-based care?	Yes<br>No<br>Unknown	Select your answer
129	If yes Q128, check all that apply	Community management of diarrhoea<br>Community management of pneumonia with antibiotics by CHWs<br>Community management of fever<br>Community management of severe malnutrition<br>Integrated community case management of diarrhoea, pneumonia, fever, and malnutrition<br>Home visits for newborn health in the postnatal period<br>Home visits for children beyond the newborn period	Check all that apply<br>☐<br>☐<br>☐<br>☐<br>☐<br>☐<br>☐
130	Which cadres of health workers provide care for sick children either at home (through home visits) or in the community (at heath posts or at the CHW's home)? (Check all that apply)	Unpaid community volunteer<br>Paid community health worker<br>Traditional birth attendant<br>Nurse<br>Physicians' assistant<br>Other	Check all that apply<br>☐<br>☐<br>☐<br>☐<br>☐<br>☐
131	What is the minimal level of education required for CHWs providing care for newborns and children in the community?	None<br>Minimal literacy and numeracy<br>Primary school<br>Secondary/high school<br>Other	Select your answer
132	Is there pre-service training for CHWs who care for newborns and/ or children in the community?	<1 month<br>1-3 months<br>3 – 6 months<br>>6 months<br>Other/unknown	Select your answer

For peer review only

133	Do CHWs receive any incentives or remuneration?	• Non-monetary incentives (e.g. rainboots, raincoat, phone credit, bicycle, etc)<br>• User fees for service<br>• A small commission on sale of medicines and other commodities supplied that CHWs can take as renumeration<br>• Salary<br>• Incentive payments (e.g. results/performance based stipend, per diem)<br>• No incentives nor remunerations	Select your answer
134	What size population is each CHW caring for newborns and children at home or in the community expected to provide care for?	< 1000 pop.<br>1000 pop.<br>> 1000 pop.<br>> 5000 pop.<br>Unknown / not defined	Select your answer

DEFINITIONS

* **IMCI implementation**

IMCI is said to be implemented if:
1) IMCI is included in national and district work plans and budgets,
2) District health facilities use the IMCI strategy to provide care for the sick child (with or without C-IMCI)

For peer review only

IMPLEMENTATION			
Srl no	Question	Responding options	Mark answers below
QUALITY OF CARE IN FIRST LEVEL HEALTH FACILITIES			
201	What year approximately did the first national IMCI training take place?	Report the year	
202	Proportion of districts having initiated IMCI training for first-level health workers	<25%<br>25 to 49%<br>50 to 74%<br>75% or more<br>unknown	Select your answer
203	Proportion of first-level health facilities with at least two health workers who care for sick children under five trained in IMCI	<25%<br>25 to 49%<br>50 to 74%<br>75% or more<br>unknown	Select your answer
204	Proportion of first-level health facilities with at least 60% of health workers who care for sick children under five trained in IMCI	<25%<br>25 to 49%<br>50 to 74%<br>75% or more<br>unknown	Select your answer
205a	What is the average duration of IMCI in-service training for doctors ?	< 5 days<br>5 – 7 days<br>7 – 10<br>11 days<br>other	Select your answer
205b	What is the average duration of IMCI in-service training for nurses and other paramedics?	< 5 days<br>5 – 7 days<br>7 – 10<br>11 days<br>other	Select your answer
206	What is the proportion of time spent on clinical practice during in-service training?	< 25%<br>25 – 40%<br>40 – 60%<br>Other ____________________	Select your answer
207	Which types of in-service IMCI training courses are currently used for training first level health workers?	Standard 11-day IMCI course<br>Short abridged IMCI course<br>IMCI-Distance learning course<br>ICATT (computer-based course) - classroom mode<br>ICATT distance learning<br>Other ________________________	Select your answer
208	Has IMCI been introduced in pre-service education?	Yes<br>No<br>Unknown	Select your answer
	If yes,		
209	What proportion of Nursing/midwifery training institutions have introduced IMCI in pre-service teaching?	<25%<br>25 to 49%<br>50 to 74%<br>75% or more<br>unknown	Select your answer
210	What proportion of medical training institutions have introduced IMCI in pre-service teaching?	<25%<br>25 to 49%<br>50 to 74%<br>75% or more<br>unknown	Select your answer

For peer review only

211	What proportion of medical officers /clinical officers training institutions have introduced IMCI in pre-service teaching?	<25%<br>25 to 49%<br>50 to 74%<br>75% or more<br>unknown	Select your answer
212	Proportion of first-level health facilities that have received at least one supervisory visit in the last six months	<25%<br>25 to 49%<br>50 to 74%<br>75% or more<br>unknown	Select your answer
QUALITY OF CARE IN REFERRAL LEVEL HEALTH FACILITIES			
213	Has the country adapted the Pocket Book for Hospital care for children guidelines in referral health facilities?	Yes<br>No<br>Unknown	Select your answer
214	When was the last update of the national paediatric guidelines?	Report the year	
215	What proportion of hospitals have introduced emergency triage assessment and treatment (ETAT)?	<25%<br>25 to 49%<br>50 to 74%<br>75% or more<br>unknown	Select your answer
216	Has the country conducted assessments of quality of paediatric hospital care?	Yes<br>No<br>Unknown	Select your answer
217	If yes, when was the last time it was done?	Report the year	
218	Does the ministry of health have a paediatric quality of care improvement program for the health facilities?	Yes<br>No<br>Unknown	Select your answer
QUALITY OF CARE IN COMMUNITY SERVICES			
219	Proportion of districts implementing integrated community case management of childhood illness	<25%<br>25 to 49%<br>50 to 74%<br>75% or more<br>unknown<br>not applicable	Select your answer
220	Proportion of districts implementing home visits for newborn health	<25%<br>25 to 49%<br>50 to 74%<br>75% or more<br>unknown<br>not applicable	Select your answer
221	Proportion of communities with a CHW trained to provide community case management of childhood illness	<25%<br>25 to 49%<br>50 to 74%<br>75% or more<br>unknown<br>not applicable	Select your answer
222	Proportion of communities with a CHW trained to conduct home visits for newborn health	<25%<br>25 to 49%<br>50 to 74%<br>75% or more<br>unknown<br>not applicable	Select your answer

For peer review only

223	Who provides supervision to CHWs? (Check all that apply)	IMCI trained supervisors<br>Non-IMCI trained supervisors<br>Peer support<br>Mentorship in health facilities	Check all that apply<br>☐<br>☐<br>☐<br>☐
225	How do CHWs facilitate referral? (Check all that apply)	Referral note<br>Phone communication with referral centre<br>Arranging transport<br>Other<br>None of the above	Check all that apply<br>☐<br>☐<br>☐<br>☐<br>☐
HEALTH SYSTEM STRENGTHENING			
226	Does the national Health Management Information System (HMIS) include monitoring indicators for IMCI	Yes<br>No<br>Unknown	Select your answer
227	If yes, at what levels are these indicators regularly collected?	Referral level<br>Primary health care level<br>Community level<br>None<br>Unknown	Select your answer
228	Is there a comprehensive monitoring & evaluation plan for IMCI?	Yes<br>No<br>Unknown	Select your answer
229	When was the most recent IMCI Health Facility Survey (HFS) conducted?	Report the year	
230	When was the most recent Child health Programme Review conducted?	Report the year	
231	Has Child Health Programme Management training been introduced?	Yes<br>No<br>Unknown	Select your answer
232	If yes, how many programme managers at all levels have been trained?	Number ________________<br>Unknown	
233	If yes, is the methodology used for annual review and planning at district level?	Yes<br>No<br>Unknown	Select your answer
234	Have tools for bottleneck analysis and strategic planning been introduced?	Yes<br>No<br>Unknown	Select your answer
235	If yes in Q234 specify the tools (check all that apply)	One Health<br>Marginal Budgeting for Bottlenecks<br>DIVA (Diagnose, Intervene, Verify & Approach)<br>EQUIST (Equitable Impact Sensitive Tool)	Check all that apply<br>☐<br>☐<br>☐<br>☐
	Does the national Essential Medicines list include the following	Amoxicillin<br>Ampicillin (Injectable)<br>Artemisinin combination therapy<br>Artesunate (Parental)<br>Benzyl penicillin<br>Ceftriaxone	Check all that apply<br>☐<br>☐<br>☐<br>☐<br>☐<br>☐

For peer review only

236	Does the national Essential Medicines list include the following medicines for use in children <5years? (Check all that apply)	Ciprofloxacin<br>Cotrimoxazole<br>Gentamycin<br>New standard/Low osmolarity ORS<br>Rectal artesunate<br>Salbutamol inhaler<br>Zinc tablets	☐<br>☐<br>☐<br>☐<br>☐<br>☐<br>☐
INNOVATIONS			
237	Have technological (mHealth, eHealth, ICT) innovations been implemented with respect to:		
	A) Behaviour change communication (BCC)	None<br>Exploration<br>Partial scale-up<br>Nationwide scale-up	Select your answer
		If implemented , specify which technology	
	B) Data collection and reporting	None<br>Exploration<br>Partial scale-up<br>Nationwide scale-up	Select your answer
		If implemented , specify which technology	
	C) Electronic decision support (protocols, algorithms, checklists)	None<br>Exploration<br>Partial scale-up<br>Nationwide scale-up	Select your answer
		If implemented , specify which technology	
	D) Provider training & education	None<br>Exploration<br>Partial scale-up<br>Nationwide scale-up	Select your answer
		If implemented , specify which technology	
	E) Supply chain management	None<br>Exploration<br>Partial scale-up<br>Nationwide scale-up	Select your answer
		If implemented , specify which technology	
	F) Financial transactions & incentives	None<br>Exploration<br>Partial scale-up<br>Nationwide scale-up	Select your answer
		If implemented , specify which technology	
VALUE OF INTEGRATED CASE MANAGEMENT OF CHILDHOOD ILLNESS			
			Check all that apply
238	What are the major strengths IMCI has brought to child health programming? Check all that apply	Quality of health services<br>Efficiency in programming<br>Efficiency in service provision<br>Cost-savings<br>Rational use of medicines<br>Holistic approach to the child	☐<br>☐<br>☐<br>☐<br>☐<br>☐

For peer review only

		Equity in access and coverage of interventions	☐
		Other (specify) ____________________	☐
BARRIERS TO IMPLEMENTATION			
			Check all that apply
239	What are some barriers to implementation of IMCI at national level ? (Check all that apply)	Strategic planning	☐
		Programme management	☐
		Budget for training	☐
		Medicine procurement & supply chain management	☐
		Mentorship and supervision	☐
		Political support and ownership	☐
		Availability of dedicated budget line in health sector plan	☐
		Cost of program / sustainability	☐
		Adaptation to new guidelines	☐
		Scaling up in-service training	☐
		Coordination and collaboration with other child health related programmes	☐
		Other (specify)_______	☐
240	What are some barriers to implementation of IMCI at regional/district level ? (Check all that apply)	Programme management	☐
		Budget for training	☐
		Medicine procurement & supply chain management	☐
		Mentorship and supervision	☐
		Staff turn over	☐
		Cost of program	☐
		Other (specify) ________	☐
241	What are some barriers to implementation of IMCI at health facility level? (Check all that apply)	Training	☐
		Mentorship and supervision	☐
		Referral links	☐
		Supply chain management	☐
		Quality of care	☐
		Staff motivation	☐
		Staff retention	☐
		Remuneration & incentives for health workers	☐
		Lack of guidelines	☐
		Lack of adherence to IMCI guidelines	☐
		Lack of enforcement of implementation of IMCI guidelines and job aids	☐
		Other (specify) _______	☐
			Check all that apply
242	What are some barriers to implementation of IMCI at the community level? (Check all that apply)	Training	☐
		Mentorship and supervision	☐
		Referral links	☐
		Supply chain management	☐
		Quality of care	☐
		Staff motivation	☐
		Staff retention	☐
		Remuneration & incentives for health workers	☐

For peer review only

		Lack of guidelines	☐
		Other (specify) ______________________________	☐

DEFINITIONS

* **IMCI implementation** IMCI is said to be implemented if:
1) IMCI is included in national and district work plans and budgets,
2) District health facilities use the IMCI strategy to provide care for the sick child (with or without C-IMCI)
3) Providers are trained in IMCI to care for the sick child at national and district levels.

For peer review only

ORGANIZATION AT NATIONAL LEVEL

Srl no	Question	Responding options	Mark answers below
301	Which unit or department is responsible for IMCI in the Ministry of Health?	Family and reproductive health<br>Maternal and child health<br>Child health<br>Community health<br>CDD/ARI<br>IMCI department / programme<br>Other (specify)<br>Not applicable	Select your answer
302	Is the same unit responsible for community health services? (if applicable)	Yes<br>No<br>Unknown	Select your answer
303	Is there a focal point forIMCI at the national level?	Yes<br>No<br>Unknown	Select your answer
304	Is there a focal point for community IMCI at the national level?	Yes<br>No<br>Unknown	Select your answer
305	Are there focal points for IMCI at district/regional level?	Yes<br>No<br>Unknown	Select your answer
306	For IMCI implementation at first-level facilities, who is the primary funder of:		
	A) Training and per diems	Government<br>Bi-lateral/multi-lateral agency* (*see definition below)<br>Other	Select your answer
	B) Medicines and equipment	Government<br>Bi-lateral/multi-lateral agency* (*see definition below)<br>Other	Select your answer
	C) Health worker salaries	Government<br>Bi-lateral/multi-lateral agency* (*see definition below)<br>Other	Select your answer
	D) Monitoring and supervision	Government<br>Bi-lateral/multi-lateral agency* (*see definition below)<br>Other	Select your answer
	E) General programme support (infrastructure, transport)	Government<br>Bi-lateral/multi-lateral agency* (*see definition below)<br>Other	Select your answer
307	For community health services who is the primary funder of:		
	Training and per diems	Government<br>Bi-lateral/multi-lateral agency* (*see definition below)<br>Other	Select your answer
	Medicines and equipment	Government<br>Bi-lateral/multi-lateral agency* (*see definition below)<br>Other	Select your answer
	Community health worker salaries	Government<br>Bi-lateral/multi-lateral agency* (*see definition below)<br>Other	Select your answer

For peer review only

	Monitoring and supervision	Government<br>Bi-lateral/multi-lateral agency* (*see definition below)<br>Other	Select your answer
	General programme support (infrastructure,	Government<br>Bi-lateral/multi-lateral agency* (*see definition below)<br>Other	Select your answer
308	Overall, what % of funding for child health would you estimate comes from internal government sources?	<25%<br>25-50%<br>50-75%<br>>75%<br>Other / unknown	Select your answer
FINAL THOUGHTS			
309	What would make access, quality and coverage of child health services stronger in your country? Please specify …		
310	Have any initiatives been implemented in your country to improve the access, quality and coverage of child health services that have not yet been referred to. Please specify …		
311	How could tools available to implement the IMCI strategy be improved? Please specify ….<br>sas		
312	If you were asked to improve IMCI and make it more relevant to today's needs, what would you suggest? Please specify …..		

DEFINITIONS

* **IMCI implementation**

IMCI is said to be implemented if:
1) IMCI is included in national and district work plans and budgets,
2) District health facilities use the IMCI strategy to provide care for sick children (with or without C-IMCI)
3) Providers are trained in IMCI to care for the sick child at national and district levels.

* **Bi-lateral agency**

A government agency or non-profit organizationbased in a single country while the agency provides aid, including medical aid or disaster relief, for people in other countries.

Examples: USAID, GIZ, JICA, the Gates Foundation, Save the Children, etc.

* **Multi-lateral agency**

International institutions with 3 or more governmental members which conduct activities in favour of development in aid recipient countries

Examples: Unicef, the World Bank, WHO

For peer review only

STROBE Statement—checklist of items that should be included in reports of observational studies

	Item No	Recommendation	Action
**Title and abstract**	1	(*a*) Indicate the study's design with a commonly used term in the title or the abstract	Yes (Title; Page 1)
		(*b*) Provide in the abstract an informative and balanced summary of what was done and what was found	Yes (Pages 1 and 2)
**Introduction**			
Background/rationale	2	Explain the scientific background and rationale for the investigation being reported	Yes (Page 3)
Objectives	3	State specific objectives, including any prespecified hypotheses	Yes (Introduction, Page 3)
**Methods**			
Study design	4	Present key elements of study design early in the paper	Yes (First sentence of Methods, Page 3)
Setting	5	Describe the setting, locations, and relevant dates, including periods of recruitment, exposure, follow-up, and data collection	Yes (within context of this survey with countries as units of analysis – no recruitment or individual level analysis)
Participants	6	(*a*) *Cohort study*—Give the eligibility criteria, and the sources and methods of selection of participants. Describe methods of follow-up<br>*Case-control study*—Give the eligibility criteria, and the sources and methods of case ascertainment and control selection. Give the rationale for the choice of cases and controls<br>*Cross-sectional study*—Give the eligibility criteria, and the sources and methods of selection of participants	Not applicable - Human subjects not directly involved in the study
		(*b*) *Cohort study*—For matched studies, give matching criteria and number of exposed and unexposed<br>*Case-control study*—For matched studies, give matching criteria and the number of controls per case	Same as above
Variables	7	Clearly define all outcomes, exposures, predictors, potential confounders, and effect modifiers. Give diagnostic criteria, if applicable	Not applicable as this was a cross-sectional survey
Data sources/ measurement	8*	For each variable of interest, give sources of data and details of methods of assessment (measurement). Describe comparability of assessment methods if there is more than one group	Yes (Methods, Page 3 containing all detailed sources of data)
Bias	9	Describe any efforts to address potential sources of bias	Yes within context of survey design (Discussion - paragraph on limitations; Page 9)
Study size	10	Explain how the study size was arrived at	Yes within context of a

For peer review only - http://bmjopen.bmj.com/site/about/guidelines.xhtml
For peer review only

			survey – no sample calculations (Methods; Page 3)
Quantitative variables	11	Explain how quantitative variables were handled in the analyses. If applicable, describe which groupings were chosen and why	Yes (Methods, Page 3)
Statistical methods	12	(*a*) Describe all statistical methods, including those used to control for confounding	Not applicable
		(*b*) Describe any methods used to examine subgroups and interactions	Not applicable
		(*c*) Explain how missing data were addressed	Not applicable
		(*d*) *Cohort study*—If applicable, explain how loss to follow-up was addressed<br>*Case-control study*—If applicable, explain how matching of cases and controls was addressed<br>*Cross-sectional study*—If applicable, describe analytical methods taking account of sampling strategy	Not applicable
		(*e*) Describe any sensitivity analyses	None performed
**Results**			
Participants	13*	(a) Report numbers of individuals at each stage of study—eg numbers potentially eligible, examined for eligibility, confirmed eligible, included in the study, completing follow-up, and analysed	Yes, in the context of countries as unit of analysis (Methods, Page 3)
		(b) Give reasons for non-participation at each stage	Yes, same as above
		(c) Consider use of a flow diagram	Yes, same as above
Descriptive data	14*	(a) Give characteristics of study participants (eg demographic, clinical, social) and information on exposures and potential confounders	Yes within context of a survey with countries as unit of analysis (Web Appendix; Table 1)
		(b) Indicate number of participants with missing data for each variable of interest	
		(c) *Cohort study*—Summarise follow-up time (eg, average and total amount)	Not applicable
Outcome data	15*	*Cohort study*—Report numbers of outcome events or summary measures over time	-
		Case-control study—Report numbers in each exposure category, or summary measures of exposure	-
		Cross-sectional study—Report numbers of outcome events or summary measures	Yes (Results; Pages 4-8)
Main results	16	(*a*) Give unadjusted estimates and, if applicable, confounder-adjusted estimates and their precision (eg, 95% confidence interval). Make clear which confounders were adjusted for and why they were included	Not applicable
		(*b*) Report category boundaries when continuous variables were categorized	Not applicable
		(*c*) If relevant, consider translating estimates of relative risk into absolute risk for a meaningful time period	Not relevant
Other analyses	17	Report other analyses done—eg analyses of subgroups and interactions, and sensitivity analyses	Qualitative analysis of open-ended questions

For peer review only - http://bmjopen.bmj.com/site/about/guidelines.xhtml

			(Results; Page 7)
**Discussion**			
Key results	18	Summarise key results with reference to study objectives	Yes (Discussion; Pages 8 and 9)
Limitations	19	Discuss limitations of the study, taking into account sources of potential bias or imprecision. Discuss both direction and magnitude of any potential bias	Yes (Page 9)
Interpretation	20	Give a cautious overall interpretation of results considering objectives, limitations, multiplicity of analyses, results from similar studies, and other relevant evidence	Yes (Page 9)
Generalisability	21	Discuss the generalisability (external validity) of the study results	Not applicable in the context of this study
**Other information**			
Funding	22	Give the source of funding and the role of the funders for the present study and, if applicable, for the original study on which the present article is based	Yes (Page 10)

*Give information separately for cases and controls in case-control studies and, if applicable, for exposed and unexposed groups in cohort and cross-sectional studies.

Note: An Explanation and Elaboration article discusses each checklist item and gives methodological background and published examples of transparent reporting. The STROBE checklist is best used in conjunction with this article (freely available on the Web sites of PLoS Medicine at http://www.plosmedicine.org/, Annals of Internal Medicine at http://www.annals.org/, and Epidemiology at http://www.epidem.com/). Information on the STROBE Initiative is available at www.strobe-statement.org.

For peer review only

BMJ Open

Global implementation survey of Integrated Management of Childhood Illness (IMCI) – twenty years on

Journal:	*BMJ Open*
Manuscript ID	bmjopen-2017-019079.R2
Article Type:	Research
Date Submitted by the Author:	28-Nov-2017
Complete List of Authors:	Boschi-Pinto, Cynthia; WHO, Maternal, Newborn, Child and Adolescent Health; Universidade Federal Fluminense, Instituto de Saúde Coletiva<br>Labadie, Guilhem; WHO, Maternal, Newborn, Child and Adolescent Health<br>Dilip, Thandassery; World Health Organization, Maternal, Newborn, Child and Adolescent Health;<br>Oliphant, Nicholas; The Global Fund to Fight AIDS, Tuberculosis, and Malaria (Geneva, Switzerland), Public Health Monitoring and Evaluation ; UNICEF, NY<br>Dalglish, Sarah; World Health Organization, Maternal, Newborn, Child and Adolescent Health<br>Aboubaker, Samira; World Health Organization, Maternal, Newborn, Child and Adolescent Health<br>AGBODJAN-PRINCE, Olga; World Health Organization, Regional Office for Africa<br>Desta, Teshome; World Health Organization, Inter-country Support Team for East and Southern Africa<br>Habimana, Phanuel; World Health Organization/Regional Office for Africa,<br>Butrón-Riveros, Betzabé ; World Health Organization, Regional Office for the Americas<br>Al-Raiby, Jamela; World Health Organization, Regional Office for Eastern Mediterranean<br>Siddeeg, Khalid; World Health Organization, Regional Office for the Eastern Mediterranean<br>Kuttumuratova, Aigul ; World Health Organization, Regional Office for Europe<br>Weber, Martin; World Health Organization, Regional Office for Europe<br>Mehta, Rajesh; WHO Regional Office for South-east Asia<br>Neena, Raina; WHO Regional Office for South-east Asia<br>DAELMANS, Bernadette; World Health Organization, Chils and Adolescent Health and Development<br>Diaz, Theresa; World Health Organization, Maternal, Newborn, Child and Adolescent Health; UNICEF, NY
**Primary Subject Heading**:	Global health
Secondary Subject Heading:	Health policy, Paediatrics, Public health
Keywords:	Child Health, IMCI, Strategic Review, IMCI Global Survey

For peer review only

Global implementation survey of Integrated Management of Childhood Illness (IMCI) – twenty years on

Authors

Cynthia Boschi-Pinto[a,b], Guilhem Labadie[a], Thandassery Ramachandran Dilip[a], Nicholas Oliphant[c,d], Sarah L. Dalglish[a], Samira Aboubaker[a], Adjoa Agbodjan-Prince[e], Teshome Desta[f], Phanuel Habimana[g], Betzabe Butron-Riveros[h], Jamela Al-Raiby[i], Khalid Siddeeg[i], Aigul Kuttumuratova[j], Martin Weber[j], Rajesh Mehta[k], Neena Raina[k], Bernadette Daelmans[a], Theresa Diaz[a]

[a] Department of Maternal Newborn Child and Adolescet Heath, World Health Organization
[b] Instituto de Saúde Coletiva, Universidade Federal Fluminense
[c] The Global Fund to Fight AIDS, Tuberculosis, and Malaria (current)
[d] UNICEF during the development of the survey (previous)
[e] World Health Organization, Regional Office for Africa, Ouagadougou, Burkina Faso
[f] World Health Organization, Regional Office for East and Southern Africa, Harare, Zimbabwe
[g] World Health Organization, Regional Office for Africa, Brazzaville, Congo
[h] World Health Organization, Regional Office for the Americas
[i] World Health Organization, Regional Office for Eastern Mediterranean
[j] World Health Organization, Regional Office for Europe
[k] World Health Organization, Regional Office for South-east Asia

Corresponding author: Cynthia Boschi-Pinto
World Health Organization, department of Maternal Newborn Child and Adolescent Health
Avenue Appia 20, 1211 Geneva, Switzerland
Email address: pintoc@who.int
Phone number: +41227913453
Fax number: +41227914853

For peer review only

Abstract

Objective: To assess the extent to which Integrated Management of Childhood Illnesses (IMCI) has been adopted and scaled up in countries.

Setting: The 95 countries that participated in the survey are home to 82% of the global under-five population and account for 95% of the 5.9 million deaths that occurred among children less than five years of age in 2015; 93 of them are low- and middle-income countries (LMICs).

Methods: We conducted a cross-sectional self-administered survey. Questionnaires and data analysis focused on 1) giving a general overview of current organization and financing of IMCI at country level; 2)

For peer review only - http://bmjopen.bmj.com/site/about/guidelines.xhtml

describing implementation of IMCI's three original components; and 3) reporting on innovations, barriers and opportunities for expanding access to care for children. A single data file was created using all information collected. Analysis was performed using STATA version 11.

Participants: In-country teams consisting of representatives of the ministry of health (MoH) and country offices of the World Health Organization (WHO) and United Nations Emergency Fund for Children (UNICEF).

Results: Eighty-one percent of countries reported that IMCI implementation encompassed all three components. Almost half (46%; 44 countries) reported implementation in 90% or more districts as well as all three components in place (full implementation). These full implementer countries were 3.6 [95% CI: 1.5-8.9] times more likely to achieve MDG-4 than other (not full implementer) countries. Despite these high reported implementation rates, the strategy is not reaching the children who need it most, as implementation is lowest in high mortality countries (39%; 7/18).

Conclusion: This survey provides a unique opportunity to better understand how implementation of IMCI has evolved in the 20 years since its inception. Results can be used to assist in formulating strategies, policies and activities to support improvements in the health and survival of children and to help achieve the health-related, post-2015 Sustainable Development Goals (SDGs).

Article Summary

Strengths and limitations of this study

This survey provides a unique opportunity to better understand how implementation of Integrated Management of Childhood Illness (IMCI) has evolved in the 20 years since its inception and help steer the direction of future child health strategies.

Limitations arising from study design include the fact that respondents may not feel comfortable providing answers that present the country in an unfavourable manner and therefore may not provide accurate answers.

The WHO African region is overrepresented in the survey responses, but disaggregation by mortality level and income groups reduces the effect of this overrepresentation on interpretation of results.

For peer review only - http://bmjopen.bmj.com/site/about/guidelines.xhtml

Introduction

WHO and UNICEF introduced Integrated Management of Childhood Illness (IMCI) in the mid-1990s as a strategy to improve child survival in countries with more than 40 deaths per 1000 live births and provide integrated prevention, treatment and care for the sick child (1). The strategy was later expanded to include care for sick newborns under one week of age, and has been regularly updated to reflect advancements in technical knowledge. Over 100 countries have adopted IMCI and implemented, in whole or in part, its three components: 1) improving health worker skills, 2) strengthening health systems, and 3) improving family and community practices. Evidence suggests that IMCI has contributed to reductions in child mortality over the era of the Millennium Development Goals (MDGs), and a recent Cochrane review found the strategy was associated with a 15% reduction in child mortality when activities were implemented in health facilities and communities (2).

Initial reviews of IMCI implementation, notably the 2003-05 Multi-Country Evaluation (3) and the 2003 Analytic Review (4), used in-depth looks at specific country contexts to draw broader lessons about implementation dynamics. WHO has also regularly surveyed countries about their adoption and implementation of the IMCI strategy, and has occasionally published maps showing the extent of implementation. However, it has not provided a comprehensive description of global implementation worldwide, with details on country variations, implementation barriers and facilitators, local innovations, and other particulars. Meanwhile UNICEF has run a number of surveys on countries' adoption and implementation of integrated Community Case Management (iCCM), a strategy similar to IMCI focusing on provision of care for sick children by community health workers (CHWs), however the data produced focused mainly on implementation in sub-Saharan African countries (5).

As part of the Strategic Review of Integrated Management of Childhood Illness (IMCI) (6,7), a global survey was carried out in April–June 2016 to assess the extent to which this strategy has been adopted and scaled up in countries over the past two decades. This article reports on its main results and provides an overview of the current status of global implementation of IMCI as informed by countries. The article also presents the main strengths and barriers in IMCI implementation, as stated by respondents, as well as suggested ways forward.

Methods

Study design

This was a cross sectional self-administered global survey carried out from April to June 2016.

Survey instrument

The survey instrument [Web Annex 1] was developed by WHO with inputs from UNICEF and was tested in three different WHO regions to ensure questions were clear and appropriate, and to estimate the time needed to complete the questionnaire. The final version was made available in all six UN languages (Arabic, Chinese, English, French, Spanish and Russian) and included mostly closed-ended questions. Countries identified the principal strengths of IMCI and barriers to its implementation by agreeing or disagreeing with a suggested list, and answered a few open-ended questions about future actions to sustain and improve implementation.

Variables

Questions and data analysis focused on variables providing: 1) a general overview of the current level of organization and financing of IMCI at country level; 2) a description of implementation of IMCI's three

For peer review only

original components and some additional components, such as iCCM; and 3) the main innovations, barriers and opportunities used in expanding access to care for the sick child.

Study size and participants

Questionnaires were sent to a total of 130 WHO Member States by the six WHO Regional Offices (ROs), which then implemented the survey through WHO country offices (COs) and provided support and follow-up. ROs had different approaches when sending out questionnaires to countries, reflecting specificities of each region. In the African region, questionnaires were sent to all 47 WHO member states. Four countries (Algeria, Cape Verde, Mauritius, and Seychelles) responded that they had not adopted IMCI and therefore did not participate in the survey. Responsible officers from South Sudan and São Tomé & Principe did not respond to the survey. In the WHO Region of the Americas, questionnaires were sent to all 33 low- and middle-income countries (LMICs) in the region, of which 19 (58%) responded. Four countries (Antigua & Barbuda, St Lucia, St Vincent & Grenadines and Venezuela) responded that they had not adopted IMCI; therefore 15 countries were included in the survey. Questionnaires were sent to 16 WHO countries in the Eastern Mediterranean region known to have adopted and implemented IMCI. Responses were received from 14 countries. Of the 53 countries in the WHO European region, 15 have introduced and/or implemented IMCI at different points in time and on varying scale. Survey questionnaires were sent to seven WHO member states where WHO National Professional Officers were present, and six responded (86%). In the South-East Asia region, questionnaires were sent to all 11 WHO member states, of which nine (82%) responded. Finally, questionnaires were sent to 16 low- and middle-income countries in the Western Pacific region, and 10 (69%) were included in the final detailed analysis. Responses were provided by in-country teams of IMCI experts with country experience, including varying combinations of representatives of the ministry of health (MoH) and WHO and UNICEF country offices, or in some instances just representatives of the MoH. Responses were tracked by WHO ROs to maximize return and clarify inconsistencies or omissions. Completed questionnaires were then returned from countries to WHO Headquarters.

Data source and analysis

Data from the Excel questionnaires were initially converted into an Excel database. Responses were checked for skip patterns and missing data were recoded. Clarification was sought from countries with missing data, of which some responded. Analyses included descriptive statistics to synthesize and organize information using numerical procedures (simple frequency and means in univariate and bivariate tables), and/or graphic techniques to describe the characteristics of IMCI in the respondent countries. Data analysis was disaggregated by levels of under-five mortality rates (U5MRs), gross national income (GNI) per capita, and extent of IMCI implementation. Countries were categorized into low (<40 deaths per 1000 live births), middle (40-79 deaths per 1000 live births) and high mortality (80+ deaths per 1000 live births). Countries were also classified by income level (low, middle, and high) according to current GNI per capita (2016) using World Bank data (8), and as high (>90% of districts reported to have implemented IMCI), middle (50-90% of districts reported to have implemented IMCI) and low implementers (<50% of districts reported to have implemented IMCI). Information on under-five mortality (numbers and rates) for 2015 was obtained from the United Nations Inter-agency Group for Child Mortality Estimation (UN-IGME) report on levels and trends in child mortality (9). Information on under-five population for the year 2015 was obtained from the UN Population Division (10). A single data file was created containing all information collected. Responses to pre-coded questions are presented in this paper. Analysis was performed using STATA version 11.0 software. More detailed methods are described elsewhere (11).

For peer review only - http://bmjopen.bmj.com/site/about/guidelines.xhtml

Patient Involvement

This is a self-administered key-informant survey carried out at national level and no individual patient data was included.

Results

Participants and Descriptive Data

Questionnaires were sent to 130 WHO Member States with a response rate of 80% [Web Table 1]. Of the 104 countries that responded to the survey, eight reported no IMCI in the country, leaving 96 countries for the general analysis. One country was dropped from the analysis because IMCI was not reported to be part of their national child health plan or strategy, no district was reported to be implementing IMCI and none of the three components was reported to be in place (Flow diagram). Of the 95 countries retained for final analysis (Figure 1), only two (Oman and Uruguay) are classified as high-income countries according to the World Bank; 64 are classified as middle-income and 29 as low-income. These 95 countries are home to 82% of the global under-five population and account for 95% of the 5.9 million deaths that occurred among children less than five years of age in 2015.

The instruction that questionnaires be completed by a three-person in-country team (from MoH, WHO and UNICEF) was followed in only five countries. In the majority of countries only the MoH provided answers [Web Table 2]. For three countries, information on respondents is not available.

Eighty-nine per cent (82 countries) of the 94 responding countries had an IMCI focal point at national level, whereas only 68% (62/91) had one at the sub-national (regional or district) level. Among high mortality countries, 88% (17/20) had a national focal point, but less than half this proportion (42%; 8/19) had subnational focal points. The presence of regional/district focal points was much higher in countries with U5MR below 80 per 1000 live births (75%; 54/72) than in countries with higher U5MR. The government is generally reported to be the principal funding source for IMCI expenditures at first level health facilities and in the community, except for health worker training and per diems, for which donors constituted the main sources of funding. In Figure 2 we show the proportion of countries reporting government as the primary funder of different elements of IMCI in first-level health facilities by under-five mortality level.

Main Results

For the purpose of the survey, IMCI is said to be implemented if it is included in national and district work plans and budgets, if district health facilities use the strategy to provide care for the sick child (with or without community-based IMCI), and if providers are trained in IMCI at national and district levels. Of the 95 countries included in the full analysis, 81 responded to the question on the proportion of districts implementing IMCI. Of these 81 responding countries, 51 (63%) reported implementing IMCI in all districts.

Figure 3 shows the proportion of countries reporting the components of IMCI they have implemented. While 92 (98%) of the 94 responding countries reported having implemented the first component of IMCI (improving health worker skills), 89 (95%) reported implementing the second (strengthening health systems), and 78 (83%) responded having implemented the third (improving family and community practices). Overall, 76 (81%) of the 94 countries reported implementing all three components.

For peer review only - http://bmjopen.bmj.com/site/about/guidelines.xhtml

Forty-four countries reported implementing IMCI in more than 90% of districts *and* having all three IMCI components in place; these are considered full implementer countries. These 44 full implementer countries were 3.6 [95% CI: 1.5 – 8.9] times more likely to achieve MDG-4 than other (not full implementer) countries, and are home to 160 million (24%) of the world's under-five children.

First component – improving health worker skills

The original training course for health workers lasted 11 days, with 30% of time spent in clinical sessions (12). Most responding countries (42%; 39/92) used shortened or abridged versions (ranging from six to eight days) of the original 11-day course. Clinical practice was at least 25% of the training time in 79% of the countries (72/91). Nearly three quarters of countries (72%; 67/93) reported implementing some sort of IMCI pre-service training.

Generic case management guidelines and related training materials were developed with the aim of being appropriate in LMICs where U5MR is higher than 40 per 1000 live births, and where there is transmission of *P. falciparum* malaria (13). Over the years, IMCI chart booklets have been continuously adapted to the context and changing scenarios of each country. Since the last global update in 2014, 81% (69/85) of countries reported having updated their national chart booklets. Recognizing the increased proportional burden of newborn mortality, nearly all countries (95%; 89/94) adapted IMCI guidelines to include care of the sick newborn in the first week of life. Development or revision of national treatment guidelines based on the 2013 revision of the WHO Pocket Book of Hospital Care for Children and its adaptation in referral facilities were reported by 55% (48/87) and 44% (39/88) of responding countries, respectively. Considering Emergency Triage Assessment and Treatment (ETAT), 23% of 65 responding countries reported introducing these guidelines in at least 75% of hospitals. This proportion is lower (17%) in the 41 middle and high mortality countries than in the 24 low mortality countries (38%).

The most common conditions added to the guidelines in national adaptations were jaundice in newborn babies and young infants (77%; 72/93), HIV (63%; 59/93), sore throat (59%; 55/93), and skin conditions (44%; 41/93). Other illnesses frequently included are tuberculosis and dengue haemorrhagic fever. Adaptations varied according to regional or national epidemiological profile.

Second component – strengthening health systems

Improving and sustaining the quality of care (QoC) is an integral part of strengthening health systems for paediatric patients. An assessment of quality of paediatric hospital care was conducted in 42% (39/92) of responding countries. Fifty-two per cent (34/65) of middle and high implementer countries reported having such assessment compared to only 8% (1/13) of low implementer countries. Fifty-eight per cent of the 92 responding countries reported having a paediatric QoC improvement programme for health facilities in their MoH. Countries that report improvement programmes together account for nearly 3 million (50%) of all 5.9 million under-five deaths occurring worldwide in 2015.

Supervision and monitoring are key to sustainable QoC and to strengthening the overall health system. Few countries (15%; 10/66) reported that more than 75% of first level facilities received at least one supervisory visit in the last six months; half (33/66) had less than one quarter of these facilities receiving visits. In general, monitoring of IMCI implementation has been carried out infrequently. Only 33% of countries (30/91) reported having a comprehensive IMCI monitoring and evaluation plan. Fifteen per cent (3/20) of high mortality countries reported having such a plan, while this proportion was 38% (27/71) in middle and low mortality countries. Still, more than two thirds of countries (70%; 66/94) reported that their Health Management Information System (HMIS) includes monitoring indicators for IMCI. Most of these indicators are collected at the primary health care level (83%; 54/65); only four countries reported having monitoring indicators for IMCI collected at community level.

For peer review only - http://bmjopen.bmj.com/site/about/guidelines.xhtml

In 2009, WHO launched the "Managing Programmes to Improve Child Health" course, which has been introduced in almost half (48%; 42/88) of responding countries. The number of managers trained in this course per under-five population varies widely, ranging from 1 manager per 840 thousand under-five children in Lao PDR to 1 manager for 1500 children in Kiribati – a more than 500-fold difference. Tools for bottleneck analysis and strategic planning have been introduced in 33 of the 79 responding countries (42%).

The main innovations reported by countries are related to information communication technologies (ICTs), which include eHealth, mHealth and Rapid SMS. Use of such innovations in the implementation of IMCI was reported over six categories: data collection and reporting (70%; 60/86); supply chain management (58%; 51/88); behaviour change communication (54%; 48/89); health provider training and education (54%; 46/85); electronic decision support (49%; 42/85); and financial transactions and incentives (41%; 35/86).

Third component - Improving family and community practices

Improving family and community practices was included as part of IMCI implementation by 83% (78/94) of responding countries (Figure 3). Eighty-nine per cent (48/54) of medium and high mortality countries report implementation of this component, while 75% (30/40) of low mortality countries reported implementing it. In general, activities to *promote key family practices for child health* have been implemented through home visits, social mobilization or community groups. The main delivery mechanisms used are illustrated in Figure 4. The majority of responding countries (71%; 45/63) reported that one community health worker (CHW) covered up to 1000 under-five population. Supervision for CHWs was mainly provided by IMCI trained supervisor (63%; 58/92) and through mentorship in health facilities (45%; 41/92).

Community care for children under five years of age was reported to be provided by more than three quarters (78%, 72/92) of responding countries. The proportion of countries with CHWs providing treatment for children, in general, was higher in high (85; 17/20) and medium (94%, 31/33) mortality countries as compared to low mortality countries (62%, 24/39). These variations are similar to those observed for the presence of community case management (CCM) policy in countries. Among the 72 countries that reported that CHWs provide care for children, 93% (67 countries) reported having CCM for diarrhoea. Of these, 80% (53/66) reported having the relevant written policy in place (one country did not respond). Conversely, six countries reported having the policy in place but not implementing it. Among the 72 countries where CHWs provided treatment for children, only 74% (53/72) reported that CHWs provide CCM for pneumonia. From these 53 countries, 43 (81%) reported having the relevant written policy in place. In 2012, the key elements of CCM were brought together in a package - integrated community case management (iCCM) (14), typically delivered by CHWs at the community level. It encompasses treatment for (i) childhood pneumonia with antibiotics, (ii) diarrhoea with zinc and oral rehydration salts and (iii) malaria with artemisinin combination therapy. Provision of iCCM was reported by 74% (53/72) of these responding countries.

Other aspects of community care, also supported by WHO and UNICEF, include the management of severe acute malnutrition (SAM) (15) and home visits (16). CCM for SAM was mentioned by 71% (51/72) of countries. Of the 95 countries participating in the survey, 65 reported that CHWs provide care for newborn babies. Of these countries, 91% (59/65) reported providing home visits to newborns. In comparison, 76% (55/72) provided home visits for children beyond the newborn period.

For peer review only - http://bmjopen.bmj.com/site/about/guidelines.xhtml

Strengths, Barriers, and Ways forward

The major strengths of IMCI most frequently identified by 93 responding countries were the holistic approach to the child (90%; 84 countries), the rational use of medicine (89%; 83 countries), the quality of health services (87%; 81 countries), and the efficiency of service provision (80%; 74 countries).

At national level, the reported major barriers to implementing IMCI were budget for training (85%; 78/92), mentorship and supervision (74%; 68/92), cost or sustainability of activities (63%; 58/92), and availability of a dedicated budget line (60%; 55/92). Major barriers reported at regional/district level were staff turnover (84%; 76/91), budget for training (82%; 75/91), and mentorship and supervision (74%; 67/91). Barriers reported at facility level were staff retention (80%; 73/91), mentorship and supervision (79%; 72/91), and staff motivation (74%; 67/91). High mortality and/or low income countries were more likely to report barriers related to budget for training, and medicine procurement and supply chain at national level. High implementer countries were more likely to report issues around mentorship and supervision, and less likely to report barriers on programme management and on political support and ownership.

When asked to suggest ways to strengthen child health services and make IMCI more relevant to today's needs, countries commonly advocated for better integration of current strategies into general health services, with a costed work plan and committed budget, as well as improved training at all levels, including pre-service training, and supportive supervision (helping to make things work, rather than checking to see if something went wrong) and mentoring. Health systems issues were said to limit implementation, with a strong need for a functioning supply chain, HMIS and monitoring and evaluation system. Better use of communications technologies was also suggested.

At the policy level, strategic planning, improved internal coordination (via a dedicated focal person for child health, regular review meetings, national steering committee) and effective coordination with partners to overcome fragmentation would be essential. Respondents said compliance with national guidelines and good implementation practices should also be recognized and rewarded. Community-level activities including outreach and home visits need more emphasis, as do the management of sick newborns at all levels. To achieve these goals, high-level advocacy and political support is said to be required, ensuring government leadership and ownership.

Discussion

This study has some limitations, most of which are inherent to survey design. Respondents may not feel comfortable providing answers that present the country in an unfavourable light and therefore may not provide accurate answers. Also, respondents may not always be fully aware of the situation in the country. To minimize this limitation, we provided clear instructions for questionnaires to be filled out by a three-person in-country team, but only five countries followed this instruction. Another 16 countries had questions answered by a two-respondent team. Bias due to non-response may also have occurred as respondents who chose to answer certain questions may differ from those who chose not to respond. Finally, the WHO African region is likely to be overrepresented in the survey, representing 43% of the responding countries, while the European region represents six per cent of them. Disaggregation by mortality level and income groups reduces the effect of this overrepresentation on the interpretation of results. Despite these limitations, this survey provides a unique opportunity to better understand how and in which direction the implementation of IMCI has evolved over the 20 years since its inception. It is also a unique opportunity to help steer the direction of future child health strategies.

This article summarizes the first survey assessment of IMCI implementation status in 95 responding countries worldwide, 20 years after its launch in the mid-1990s (11). In 1998, 12 countries had already

For peer review only - http://bmjopen.bmj.com/site/about/guidelines.xhtml

moved into the expansion phase of IMCI's first component and had begun introducing the other two components (17). Twenty years later, coverage of IMCI is reported to be comprehensive in many target countries; however few countries have achieved full scale-up and, in many aspects, implementation remains incomplete. Moreover, regardless of high reported implementation, the strategy is still not reaching the children who need it most, since coverage is lowest in high mortality countries. Nevertheless, it is worth noting that high implementer countries (≥90% of districts) with all three IMCI components in place are home to 160 million (24%) of the global under-five children. These full implementer countries were 3.6 [95% CI: 1.5 – 8.9] times more likely to achieve MDG-4 than other (not full implementer) countries. Also of note is the fact that among the 24 low and lower-middle income countries that achieved the MDG-4 target, 18 (75%) are full implementer countries. Our results reinforce the original concept that full implementation of IMCI can lead to a substantial impact on child health and survival.

The training course for health workers developed by WHO and UNICEF is one of the key elements of IMCI strategy. Some limited evidence suggests the original 11-day training course to be more effective than shortened training (18); however, concerns have been raised regarding the costs and the absence of health workers from their duties (19). Most countries reported using shortened or abridged versions of the original course. Flexibility to add conditions according to regional and national epidemiologic profile has facilitated adaptation of IMCI guidelines to a changing epidemiological profile. Supervision is the weakest area under the second IMCI component, with only 15% of countries reporting more than three-quarters of first level facilities receiving at least one supervisory visit in the 6 months before the survey. Similar results have recently been shown in a study carried out in Afghanistan, where only 11% of health workers received supervision during the past 6 months (20). Lack of supervision was also reported as a barrier in an overview of IMCI implementation in West Java province, Indonesia (21). The most commonly mentioned barriers must be foremost in the minds of our thinking on and re-designing the future of child health and development.

There is widespread recognition that IMCI will only result in improvements in child health and survival if training activities are accompanied by effective efforts to strengthen health systems and reach children and mothers in the community. It has also been recognized that "strengthening health systems is the best way to safeguard against health crises" (22). Sadly, results highlight the weakness of the implementation of IMCI second component. Supervision and mentorship were among the areas with the least impressive results. Special attention should be directed to the second component of IMCI The sustainability of IMCI, and of the gains in child health this strategy can help achieve, will depend on countries' efforts to make real improvements in their health systems – beginning with providing adequate funding for health worker salaries, training, and supplies, among other inputs.

Conclusion

Despite the multiple and extensive constraints in IMCI implementation reported here, the findings of this survey also provide an indication of the value and esteem that country-level stakeholders assign to IMCI. Results point to a unique opportunity to help steer future policies, programmes and strategies to promote child health. Given the many competing priorities of survey respondents, the 80% response rate obtained reveals the interest IMCI still elicits, especially in low and middle income countries, and suggests that a strengthened IMCI has a role in attending the call for "Survive, Thrive, Transform" from the Global Strategy for Women's Children's and Adolescents' Health 2016-2030 (23). IMCI has generated a paradigm shift towards integrated programming for child health globally and in countries, which should be built upon in the current era of the Sustainable Development Goals (SDGs), under which global leaders have committed to end preventable newborn and child mortality. Full implementation of IMCI in health facilities and communities with a critical focus on health systems strengthening will be decisive for countries to secure universal health care and help achieve the health-related SDGs.

For peer review only - http://bmjopen.bmj.com/site/about/guidelines.xhtml

Acknowledgements

We thank our colleagues from WHO and UNICEF Country Offices and those from the Ministries of Health for their contribution on responding to the survey questionnaires and on providing follow-up of the survey.

Disclaimer

The authors* are staff members of the World Health Organization. The authors alone are responsible for the views expressed in this publication and they do not necessarily represent the decisions or policies of the World Health Organization.

Competing Interest

All authors have completed the Unified Competing Interest form (available on request from the corresponding author) and declare: no support from any organisation for the submitted work; no financial relationships with any organisations that might have an interest in the submitted work in the previous three years, no other relationships or activities that could appear to have influenced the submitted work.

Copyright

The Corresponding Author has the right to grant on behalf of all authors and does grant on behalf of all authors, an exclusive licence (or non exclusive for government employees) on a worldwide basis to the BMJ Publishing Group Ltd to permit this article to be published in BMJ editions and any other BMJPGL products and sublicences such use and exploit all subsidiary rights, as set out in our licence.

Funding

The Strategic Review of IMCI and iCCM was funded by the Bill and Melinda Gates Foundation; the authors donated their time to writing the article; the publication fees for the supplement were funded by the Health Systems Research Unit, South African Medical Research Council.

Contributors and sources

The authors bring a combination of expertise in epidemiology, demography, statistics, child health interventions and policies, public health, and advocacy. CBP conceptualized and wrote the first drafts of the paper. GL, TRD and NO performed the analysis. GL conceptualized and performed the illustrations and revised the first draft. SD analyzed the qualitative information and wrote the last section of the paper (Strengths, barriers and ways forward). BD and SA developed the questionnaire. SA revised several versions of the paper, providing advice on IMCI-specific issues; helped coordinate the distribution and application of questionnaires and followed-up with WHO Regional Offices. AA-P, TD, PH, BB-R, JA-R, KS, AK, MW, RM, and NR organized, tested, followed-up and reviewed the questionnaires sent to countries. All authors contributed intellectual content and critical revisions to the manuscript. All authors approved the final manuscript. CBP is guarantor.

Data Sharing

All data will be available in the public domain [IMCI Global Implementation Survey Report] at: http://who.int/maternal_child_adolescent/documents/imci-global-survey-report/]

For peer review only - http://bmjopen.bmj.com/site/about/guidelines.xhtml

For peer review only

.11

References

(1) WHO (1997). *First Global Review and Coordination Meeting on Integrated Management of Childhood Illness (IMCI).* [online] Santo Domingo: World Health Organization. Available at: http://apps.who.int/iris/bitstream/10665/64629/1/WHO_CHD_97.11.pdf

(2) Gera T, Shah D, Garner P, Richardson M, Sachdev HS. Integrated management of childhood illness (IMCI) strategy for children under five. Cochrane Database Syst Rev. 2016 Jun 22;(6):CD010123. doi: 10.1002/14651858.CD010123.pub2.

(3) Bryce J, Victora CG, Habicht JP, Black RE, Scherpbier RW; MCE-IMCI Technical Advisors. Programmatic pathways to child survival: results of a multi-country evaluation of Integrated Management of Childhood Illness. *Health Policy Plan*. 2005 Dec;20 Suppl 1:i5-i17.

(4) WHO (2003b). *The analytic review of the Integrated Management of Childhood Illness strategy Final report.* Available at: http://www.who.int/maternal_child_adolescent/documents/9241591730/en/ [Accessed 17 Jun. 2017].

(5) Rasanathan K, Muñiz M, Bakshi S, Kumar M, Solano A, Kariuki W, George A, Sylla M, Nefdt R, Young M, Diaz T. Community case management of childhood illness in sub–Saharan Africa – findings from a cross–sectional survey on policy and implementation. *J Glob Health* 2014 Dec; 4(2): 020401. doi: 10.7189/jogh.04.020401 PMCID: PMC4267096

(6) WHO. "Towards a Grand Convergence for child survival and health: A strategic review of options for the future building on lessons learnt from IMNCI." Geneva: WHO, 2016. Available at: http://www.who.int/maternal_child_adolescent/documents/strategic-review-child-health-imnci/en/

(7) Dalglish et al. Methods for the Strategic Review of IMNCI and iCCM programmes BMJ 2017 (paper 2 in the supplement – TBD)

(8) World Bank list of economies (December 2016) https://datahelpdesk.worldbank.org/knowledgebase/articles/906519-world-bank-country-and-lending-groups. Accessed on 26.01.2017

(9) WHO/ The World Bank/ United Nations Population Division. Levels & Trends in Child Mortality Report 2015. Estimates developed by the UN Inter-agency Group for Child Mortality Estimates. Available at: http://www.who.int/maternal_child_adolescent/documents/levels_trends_child_mortality_2015/en/

(10) United Nations, Department of Economic and Social Affairs, Population Division (2015). World Population Prospects: The 2015 Revision, custom data acquired via website: https://esa.un.org/unpd/wpp/Download/Standard/Population/

(11) WHO. IMCI Global Implementation Survey Report. Available at: http://who.int/maternal_child_adolescent/documents/imci-global-survey-report/

(12) WHO. IMCI Planning Guide. WHO, 1999. Available at: http://apps.who.int/iris/bitstream/10665/66720/1/WHO_CHS_CAH_99.1_eng.pdf

(13) WHO/UNICEF. IMCI Information Package, 1999. Available at: http://apps.who.int/iris/bitstream/10665/65002/4/WHO_CHS_CAH_98.1D_eng.pdf

(14) UNICEF (2012) Integrated Community Case Management: WHO/UNICEF Joint Statement. UNICEF, New York.

For peer review only - http://bmjopen.bmj.com/site/about/guidelines.xhtml

(15) WHO (2007), Community-based management of Severe Acute Malnutrition: Joint statement by WHO, WFP and UNICEF. Malnutrition: Joint statement by WHO, WFP and UNICEF. WHO, Geneva.

(16) WHO/Unicef (2009) WHO/Unicef Joint Statement on Home Visits for the Newborn Child: A strategy to Improve Survival. WHO, Geneva.

(17) Tulloch J. Integrated approach to child health in developing countries. Lancet 1999;354 Suppl 2;SII16-20

(18) Rowe AK, Rowe SY, Holloway KA et al. Does shortening the training on Integrated Management of Childhood Illness guidelines reduce its effectiveness? A systematic review. *Health Policy and Planning* 2012; 27:179-93.

(19) Goga AE, Muhe LM, Forsyth K, Chopra M, Aboubaker S, Martines J, Mason EM. Results of a multi-country exploratory survey of approaches and methods for IMCI case management training. *Health Res Policy Syst* 2009; 7-18.

(20) Mansoor GF, Chikvaidze P, Varkey S, Higgins-Steele A, Safi N, Mubasher A, Yusufi K, Alawi SA. Quality of child healthcare facilities: a national assessment of the Integrated Management of Childhood Illnesses in Afghanistan. *International Journal for Quality in Health Care* 2017; 29:55-62.

(21) Titaley CR, Jusril H, Ariawan I, Soehamo N, Setiawan T, Weber MW. Challenges to the implementation of the integrated management of childhood illness (IMCI) at community health centres in West Java province, Indonesia. *WHO South Esat Asia J Public Health* 2014; 3:161-70

(22) Ghebreyesus TA. All roads lead to universal health coverage. *Lancet Glob Health* 2017; Sep; 5(9): e839-e840.doi:10.1016/ S2214-109X(17)30295-4. Epub 2017 Jul 17.

(23) Global Strategy for Women's, Children's and Adolescents Health 2016-2030. Available at: http://www.who.int/life-course/partners/global-strategy/en/

List of Figures:

Figure 1: Survey summary map

Figure 2: Proportion of countries reporting government as primary funder of various elements of IMCI in first-level health facility by under-five mortality level

Figure 3. Implementation of the three IMCI components

Figure 4. Main delivery mechanisms used for the promotion of key family practices related to child health

For peer review only - http://bmjopen.bmj.com/site/about/guidelines.xhtml

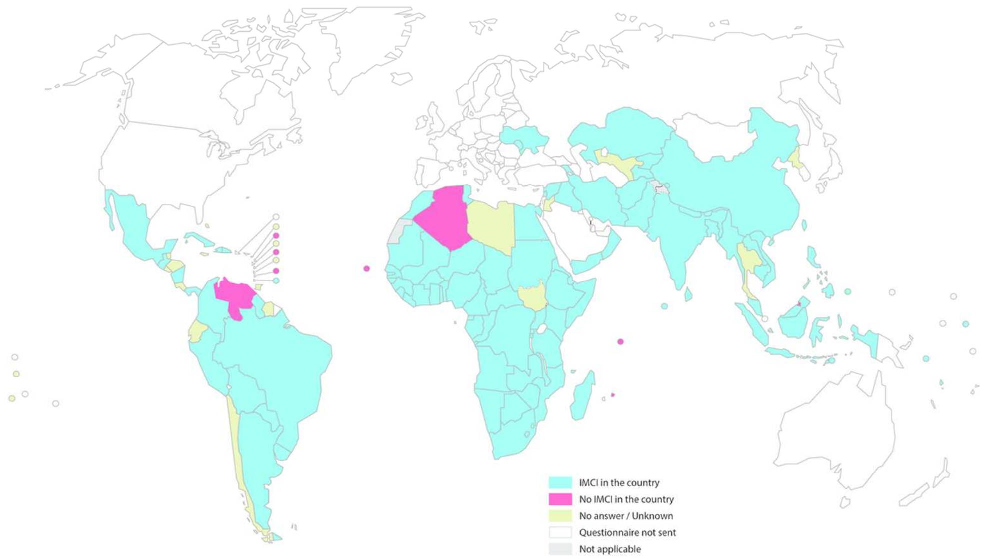

Figure 1: Survey summary map

120x69mm (300 x 300 DPI)

For peer review only

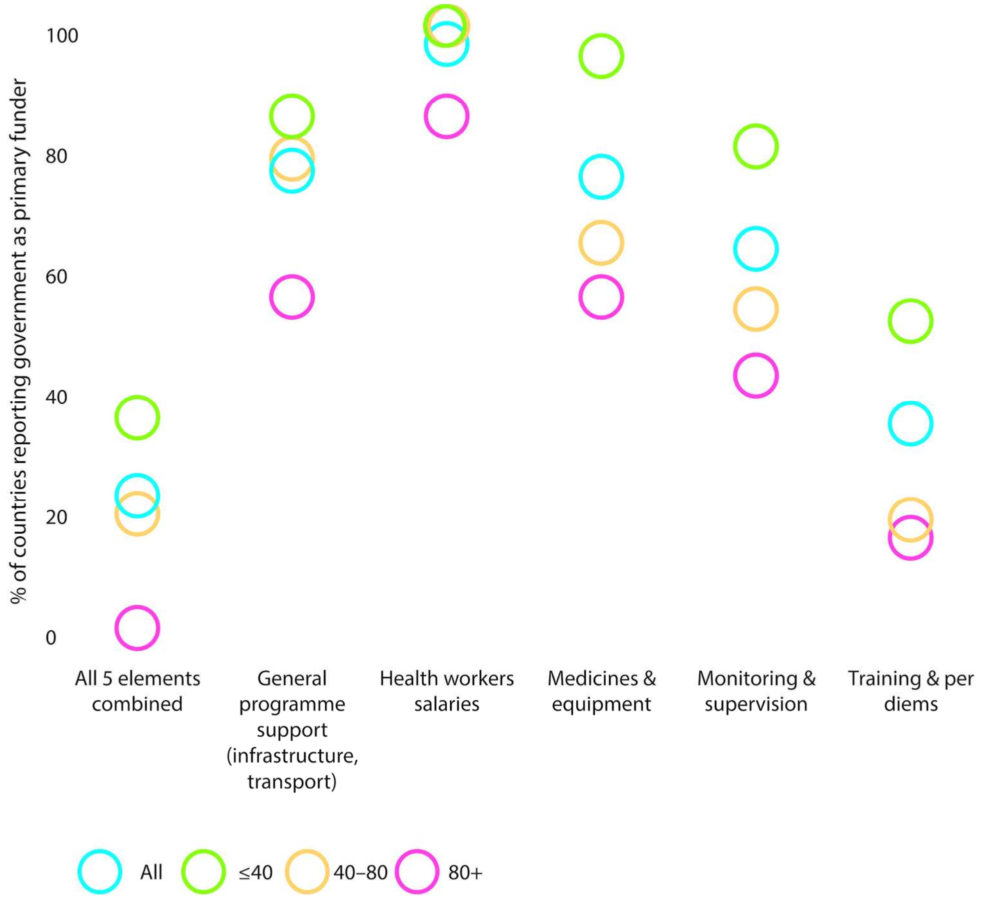

Figure 2: Proportion of countries reporting government as primary funder of various elements of IMCI in first level health facility by under-five mortality level

186x166mm (300 x 300 DPI)

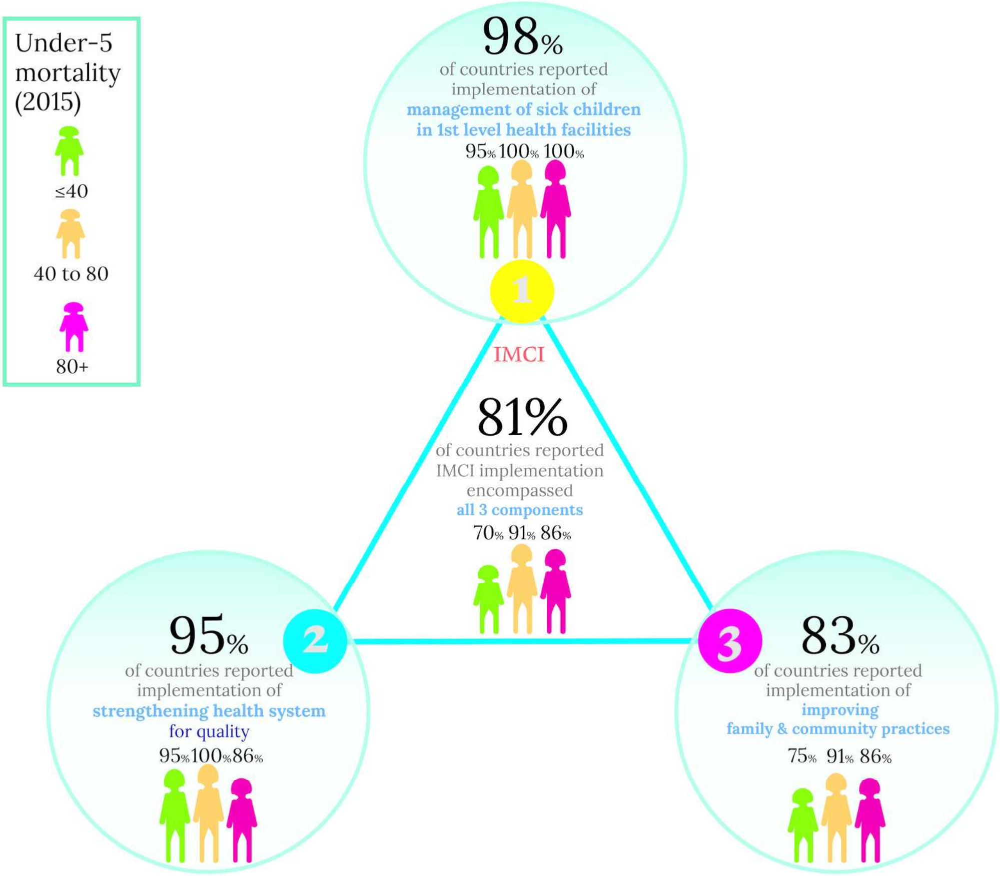

Figure 3. Implementation of the three IMCI components

209x185mm (300 x 300 DPI)

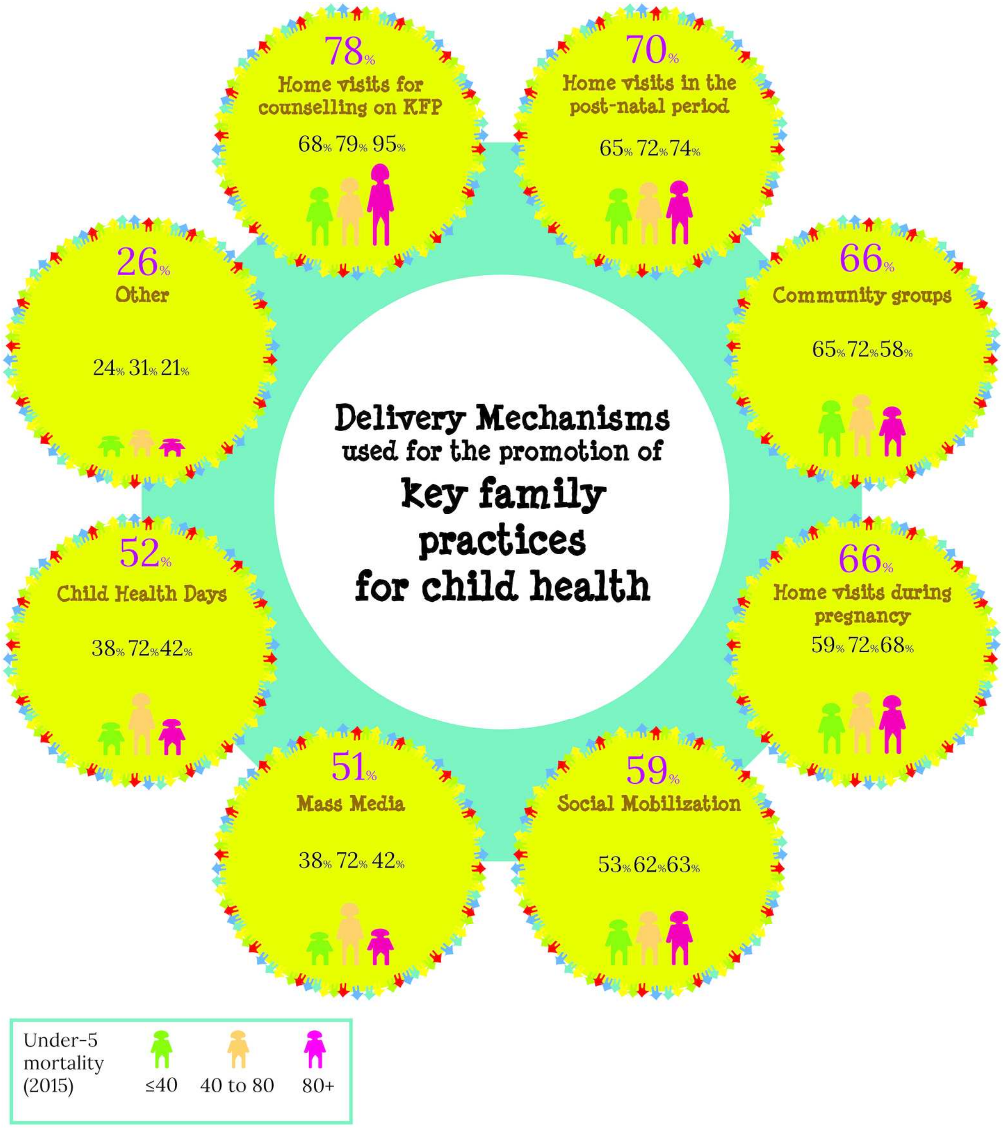

Figure 4. Main delivery mechanisms used for the promotion of key family practices related to child health

236x265mm (300 x 300 DPI)

Flow diagram

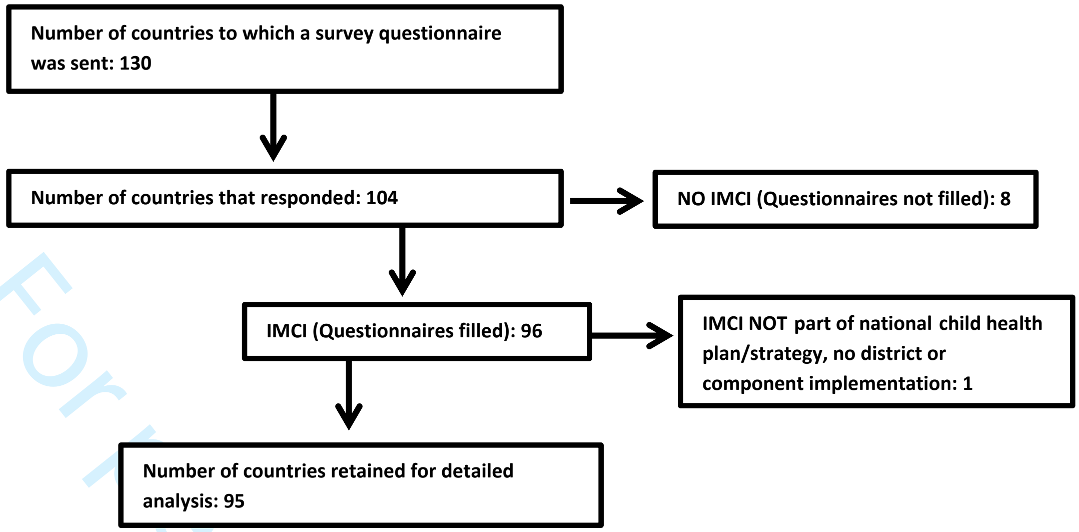

For peer review only

Web Appendix

Table 1. Response Rate to IMCI Global Implementation Survey

WHO Region	Total number of WHO MS	Member States (N) to which a survey questionnaire was sent	WHO MS that responded*	WHO MS that reported no IMCI (did not participate in the survey)	WHO MS that responded IMCI not in national plan/no district implementing nor component implemented	Response rate
**African**	47	47	45	4		95.7
**Americas**	35	33	19	4		57.6
**Eastern Mediterranean**	21	16	14			87.5
**Europe**	53	7	6			85.7
**South-east Asia**	11	11	9			81.8
**Western Pacific**	27	16	11		1	68.8
**Total**	**194**	**130**	**104**	**8**	**1**	80.0

MS = Member State; * Includes those MSs that responded not having adopted IMCI and therefore not answering the questions of the survey

Table 2. Respondents to IMCI Global Implementation Survey by WHO Region

WHO Region	Questionnaires included in final analysis	Three-team response (MoH/ WHO/ UNICEF)	Response by MoH/ WHO team	Response by MoH alone	Response by WHO alone	Response by WHO/ UNICEF team	Unknown
**African**	41	1	1	22	14	-	3
**Americas**	15	-	5	9	1	-	-
**Eastern Mediterranean**	14	3	7	4	-	-	-
**Europe**	6	1	-	3	1	1	-
**South-east Asia**	9	-	-	6	3	-	-
**Western Pacific**	10	-	1	9	-	-	-
**Total**	**95**	**5**	**15**	**52**	**19**	**1**	**3**

For peer review only

STROBE Statement—checklist of items that should be included in reports of observational studies

	Item No	Recommendation	Action
**Title and abstract**	1	(*a*) Indicate the study's design with a commonly used term in the title or the abstract	Yes (Title; Page 1)
		(*b*) Provide in the abstract an informative and balanced summary of what was done and what was found	Yes (Pages 1 and 2)
**Introduction**			
Background/rationale	2	Explain the scientific background and rationale for the investigation being reported	Yes (Page 3)
Objectives	3	State specific objectives, including any prespecified hypotheses	Yes (Introduction, Page 3)
**Methods**			
Study design	4	Present key elements of study design early in the paper	Yes (First sentence of Methods, Page 3)
Setting	5	Describe the setting, locations, and relevant dates, including periods of recruitment, exposure, follow-up, and data collection	Yes (within context of this survey with countries as units of analysis – no recruitment or individual level analysis)
Participants	6	(*a*) *Cohort study*—Give the eligibility criteria, and the sources and methods of selection of participants. Describe methods of follow-up<br>*Case-control study*—Give the eligibility criteria, and the sources and methods of case ascertainment and control selection. Give the rationale for the choice of cases and controls<br>*Cross-sectional study*—Give the eligibility criteria, and the sources and methods of selection of participants	Not applicable - Human subjects not directly involved in the study
		(*b*) *Cohort study*—For matched studies, give matching criteria and number of exposed and unexposed<br>*Case-control study*—For matched studies, give matching criteria and the number of controls per case	Same as above
Variables	7	Clearly define all outcomes, exposures, predictors, potential confounders, and effect modifiers. Give diagnostic criteria, if applicable	Not applicable as this was a cross-sectional survey
Data sources/ measurement	8*	For each variable of interest, give sources of data and details of methods of assessment (measurement). Describe comparability of assessment methods if there is more than one group	Yes (Methods, Page 3 containing all detailed sources of data)
Bias	9	Describe any efforts to address potential sources of bias	Yes within context of survey design (Discussion - paragraph on limitations; Page 9)
Study size	10	Explain how the study size was arrived at	Yes within context of a

For peer review only

			survey – no sample calculations (Methods; Page 3)
Quantitative variables	11	Explain how quantitative variables were handled in the analyses. If applicable, describe which groupings were chosen and why	Yes (Methods, Page 3)
Statistical methods	12	(*a*) Describe all statistical methods, including those used to control for confounding	Not applicable
		(*b*) Describe any methods used to examine subgroups and interactions	Not applicable
		(*c*) Explain how missing data were addressed	Not applicable
		(*d*) *Cohort study*—If applicable, explain how loss to follow-up was addressed<br>*Case-control study*—If applicable, explain how matching of cases and controls was addressed<br>*Cross-sectional study*—If applicable, describe analytical methods taking account of sampling strategy	Not applicable
		(*e*) Describe any sensitivity analyses	None performed
**Results**			
Participants	13*	(a) Report numbers of individuals at each stage of study—eg numbers potentially eligible, examined for eligibility, confirmed eligible, included in the study, completing follow-up, and analysed	Yes, in the context of countries as unit of analysis (Methods, Page 3)
		(b) Give reasons for non-participation at each stage	Yes, same as above
		(c) Consider use of a flow diagram	Yes, same as above
Descriptive data	14*	(a) Give characteristics of study participants (eg demographic, clinical, social) and information on exposures and potential confounders	Yes within context of a survey with countries as unit of analysis (Web Appendix; Table 1)
		(b) Indicate number of participants with missing data for each variable of interest	
		(c) *Cohort study*—Summarise follow-up time (eg, average and total amount)	Not applicable
Outcome data	15*	*Cohort study*—Report numbers of outcome events or summary measures over time	-
		Case-control study—Report numbers in each exposure category, or summary measures of exposure	-
		Cross-sectional study—Report numbers of outcome events or summary measures	Yes (Results; Pages 4-8)
Main results	16	(*a*) Give unadjusted estimates and, if applicable, confounder-adjusted estimates and their precision (eg, 95% confidence interval). Make clear which confounders were adjusted for and why they were included	Not applicable
		(*b*) Report category boundaries when continuous variables were categorized	Not applicable
		(*c*) If relevant, consider translating estimates of relative risk into absolute risk for a meaningful time period	Not relevant
Other analyses	17	Report other analyses done—eg analyses of subgroups and interactions, and sensitivity analyses	Qualitative analysis of open-ended questions

For peer review only

			(Results; Page 7)
**Discussion**			
Key results	18	Summarise key results with reference to study objectives	Yes (Discussion; Pages 8 and 9)
Limitations	19	Discuss limitations of the study, taking into account sources of potential bias or imprecision. Discuss both direction and magnitude of any potential bias	Yes (Page 9)
Interpretation	20	Give a cautious overall interpretation of results considering objectives, limitations, multiplicity of analyses, results from similar studies, and other relevant evidence	Yes (Page 9)
Generalisability	21	Discuss the generalisability (external validity) of the study results	Not applicable in the context of this study
**Other information**			
Funding	22	Give the source of funding and the role of the funders for the present study and, if applicable, for the original study on which the present article is based	Yes (Page 10)

*Give information separately for cases and controls in case-control studies and, if applicable, for exposed and unexposed groups in cohort and cross-sectional studies.

Note: An Explanation and Elaboration article discusses each checklist item and gives methodological background and published examples of transparent reporting. The STROBE checklist is best used in conjunction with this article (freely available on the Web sites of PLoS Medicine at http://www.plosmedicine.org/, Annals of Internal Medicine at http://www.annals.org/, and Epidemiology at http://www.epidem.com/). Information on the STROBE Initiative is available at www.strobe-statement.org.

For peer review only

For peer review only - http://bmjopen.bmj.com/site/about/guidelines.xhtml
